# Adherence to the Mediterranean and Mediterranean‐Dietary Approaches to Stop Hypertension Intervention for Neurodegenerative Delay (MIND) Diets and Parkinson's Disease Incidence in Women: Results from the Prospective E3N Cohort

**DOI:** 10.1002/ana.78115

**Published:** 2026-01-06

**Authors:** Mariem Hajji‐Louati, Emmanuelle Correia, Pei‐Chen Lee, Fanny Artaud, Emmanuel Roze, Francesca Romana Mancini, Alexis Elbaz

**Affiliations:** ^1^ Université Paris‐Saclay, UVSQ, Inserm, Gustave Roussy, CESP Villejuif France; ^2^ Department of Public Health College of Medicine, National Cheng Kung University Tainan Taiwan; ^3^ Neurology Department AP‐HP, Hôpital Pitié‐Salpêtrière Paris France; ^4^ Sorbonne Université Paris France; ^5^ INSERM U1127, CNRS 7225, Brain Institute Paris France

## Abstract

**Objective:**

The evidence regarding adherence to dietary patterns and Parkinson's disease (PD) risk is inconsistent. Because of the long prodromal PD phase, reverse causation represents a major threat to investigations of diet in relation to PD. We examined whether adherence to the Mediterranean (MED) and Mediterranean‐Dietary Approaches to Stop Hypertension Intervention for Neurodegenerative Delay (MIND) diets is associated with PD incidence, while considering reverse causation, in a large cohort of women with a 25‐year follow‐up.

**Methods:**

Participants from the E3N (Etude Epidémiologique auprès des femmes de la Mutuelle Générale de l’Education Nationale) study were prospectively followed‐up from 1993 to 2018. PD diagnoses were validated using medical records and drug claim databases. Baseline MED and MIND scores were computed using a validated food questionnaire. Hazard ratios (HR) and 95% confidence intervals (CI) were estimated using multivariable Cox regression models. Exposures were lagged by 5 years in main analyses and longer lags in sensitivity analyses. We performed age‐stratified analyses and adjusted for prodromal symptoms.

**Results:**

Analyses (5‐year–lag) are based on 71,542 women (845 PD patients). Higher adherence to MED and MIND diets was not associated with PD overall, but was associated with lower PD incidence in women <71 years old (MED, HR_high vs. low+medium_ = 0.76 [0.58–1.00], *p*‐Age × MED interaction = 0.038; MIND, HR_high vs. low+medium_ = 0.75 [0.58–0.97], *p*‐Age × MIND interaction = 0.035). Legumes and high unsaturated to saturated fat ratio had the strongest contribution for the MED diet, while beans and olive oil had the strongest contribution for the MIND diet. Results were consistent after adjustment for constipation/depression and in analyses with lags up to 20 years.

**Interpretation:**

Adherence to the MED and MIND diets was associated with lower PD incidence <71 years in women. These findings are important for planning preventative interventions. ANN NEUROL 2026;99:1014–1029

Parkinson's disease (PD) is the neurodegenerative disease with the fastest increase in prevalence. There is an urgent need to identify interventions that could prevent or delay PD onset.^1^ Inflammation, oxidative stress, and mitochondrial dysfunction are involved in PD etiology.^2^ Gut microbiota is also hypothesized to influence PD via the gut–brain axis.^3^

Previous studies suggest a relation between diet and PD.^4^ However, PD has a long prodromal phase, which may lead to reverse causation bias,^5^ because prodromal symptoms (eg, constipation, anxiety, depression, or sleep disorders) may induce dietary changes before disease onset.^6^ Prospective studies with long follow‐ups, allowing to assess diet long before disease onset, are necessary to rule out reverse causation.

Several studies examined associations between specific foods/nutrients and PD.^7^ However, foods/nutrients are not consumed in isolation. An alternative approach consists in examining the role of diet as a whole, through dietary patterns that take into account interactive and synergistic effects of foods/nutrients.^8^, ^9^

The Mediterranean (MED) and Mediterranean‐Dietary Approaches to Stop Hypertension Intervention for Neurodegenerative Delay (MIND) diets are 2 dietary patterns characterized by high antioxidant and anti‐inflammatory properties.^10^ Three prospective studies that examined the association between the MED diet and PD provided suggestive evidence of an inverse relationship.^11^, ^12^, ^13^ Only 1 study addressed reverse causation by including a 4‐year exposure lag.^11^ In addition, a meta‐analysis examining the association of the MED diet with PD, prodromal PD, and parkinsonism found that the association was stronger for those with younger onset.^14^ No prospective studies on the MIND diet and PD are available.

Our primary objective was to examine associations of the MED and the MIND diets with PD in a large French cohort of women with a 25‐year follow‐up, while carefully addressing the potential for reverse causation by implementing a 5‐year exposure lag in main analyses. As a secondary objective, we examined whether age modified the association of the MED and MIND diets with PD.

## Patients and Methods

### Etude Epidémiologique auprès des femmes de la Mutuelle Générale de l’Education Nationale Cohort Study

Etude Epidémiologique auprès des femmes de la Mutuelle Générale de l’Education Nationale (E3N) is a French prospective cohort of 98,995 women 40 to 65 years old at recruitment in 1990 and affiliated to a national health insurance plan covering people working in education.^15^ Participants complete self‐administered questionnaires every 2 to 3 years and provide information on lifestyle and health status. The average response rate at each follow‐up questionnaire is ~80%. A total of <3% of the participants have been lost to follow‐up since 1990. Since 2004, data on outpatient reimbursements of health expenditures, including drug claims, are available. Causes of death are available until 2014.

### PD Case Ascertainment

PD ascertainment is described in detail elsewhere.^16^ Briefly, potential PD patients were identified through self‐reported doctor diagnoses of PD in follow‐up questionnaires (last available, questionnaire 11 [Q11] in 2014), antiparkinsonian drug claims databases (until December 2018; Anatomical Therapeutic Chemical code, N04), and death certificates (International Classification of Disease, 332.0, G20).

Potential PD patients were contacted by mail to confirm self‐reported diagnoses. For participants who confirmed a PD or parkinsonism diagnosis and those who could not be reached, we obtained detailed medical records from treating neurologists or general practitioners. Medical records were reviewed by an expert panel to adjudicate PD status (definite, probable, possible, or no PD).^17^ Only definite and probable PD cases were retained in our analyses.

When medical records were not available, PD status was adjudicated using a validated algorithm (94% sensitivity, 88% specificity) based on antiparkinsonian drug claims and medical visits.^16^ Among participants with a final PD diagnosis, 62% of diagnoses were adjudicated based on medical records and 38% based on the algorithm.

Year of PD diagnosis was defined as the year of diagnosis according to medical records or, in decreasing order of priority, self‐reported year, year of first use of antiparkinsonian drugs, and year of the first questionnaire where PD was self‐reported.

PD incidence rates in E3N are in agreement with those observed in women from Western Europe between 1992 and 2018.^16^ This finding supports the validity of our case ascertainment strategy.

### Dietary Data

Dietary data were collected through a validated 208‐item semi‐quantitative dietary questionnaire sent in 1993 (Q3).^18^ The questionnaire assessed daily consumption of 208 food items over the past year by collecting food frequency and portion sizes for 8 meal occasions (breakfast, morning snack, appetizers before lunch, lunch, afternoon snack, appetizers before dinner, dinner, and after dinner snack). Portion sizes were estimated with the help of a photo booklet.^19^ The questionnaire's validity and reproducibility are described elsewhere.^18^ Daily intakes of energy, total unsaturated and saturated fats, and caffeine were estimated using a food composition databases derived from the French food composition table of the French Information Centre on Food Quality.^20^ Polyphenol intake was estimated using the Phenol‐Explorer database that covers many food items and polyphenols and allows a comprehensive standardized assessment.^21^

#### The MED Diet Score

We defined a score of adherence to the MED diet as a 9‐unit dietary score based on intake of fruit, vegetables, legumes, cereals, olive oil, fish, dairy products, meat products, and alcohol. To account for the low consumption of olive oil in non‐Mediterranean countries, we adopted a modified version of this score.^22^ Lipid intake was assessed by calculating the ratio of total unsaturated fats to saturated fats.^23^

Cut‐off values of 0 or 1 were assigned to each component based on the median intake among participants. For MED components commonly consumed (fruit, vegetables, legumes, cereal products, and fish) and for the ratio of unsaturated to saturated lipids, a value of 1 was given if the intake was above the median, and a value of 0 otherwise. For MED components that are traditionally less consumed (dairy, meat), a value of 0 was assigned if the intake was above the median and a value of 1 otherwise. For alcohol intake, a value of 1 was assigned to women who consumed a moderate amount (5–25g/d) and a value of 0 otherwise.

The MED score is comprised between 0 and 9, with higher scores representing greater adherence to the MED diet. The score was categorized into approximate tertiles to reflect low (0–3), medium (4, 5), and high (6–9) adherence to the MED diet.^22^

#### The MIND Diet Score

The MIND diet score includes the consumption of 15 dietary components, including 10 brain healthy components (green leafy vegetables, other vegetables, nuts, berries, beans, whole grains, fish, poultry, olive oil, and wine) and 5 unhealthy dietary components (red meats, butter and margarine, cheese, pastries/sweets, and fast fried foods).^24^ Values of 0, 0.5, or 1 are assigned to each component (except for olive oil) using fixed cut‐offs according to their frequency of consumption (higher values correspond to more frequent consumption for healthy components and less frequent consumption for unhealthy components). For olive oil, a value of 1 is assigned if it is the primary oil usually used and 0 otherwise. The sum takes values from 0 (no adherence) to 15 points (maximum adherence).

We used a French‐adapted version of the MIND diet previously developed to take into account differences in dietary habits between United States (US) and France.^25^ We replaced berry intake by total polyphenol intake. Berries were included in the original MIND score because of their high content of polyphenols, but the consumption of berries in France is much lower than in North America. Thresholds were also adapted to French dietary habits, and we used a binary (no/yes) cut‐off for components that were not frequently consumed among study participants (whole grains, nuts), and tertiles of daily intake were used for green leafy vegetables and polyphenols.

### Covariates

Data on birth date, region of residence (rural/urban), age at menarche (≤11/12–13/≥14 years), parity (nulliparous, 1 child, 2 children, and ≥3 children), menopausal status (premenopausal and natural, artificial, or unknown type of menopause), physical activity (metabolic equivalent of task hours per week [MET‐h/week]), and smoking status (never, ex, and current) were collected at baseline (Q3‐1993). Constipation (no/yes) and history of treated depression (no/yes) were created by combining information collected in Q1 (1990) and Q2 (1992) questionnaires. Weight and height were available at Q1, and body mass index (BMI) was computed as weight divided by height squared (kg/m^2^) and categorized into a 4‐level variable (underweight, <18.5; normal weight, 18.5–24.9; overweight, 25.0–29.9; obese, ≥30.0). Self‐reported history of diabetes and hypertension before Q3 were also available.

### Statistical Analysis

Analyses were conducted using SAS 9.4 (SAS Institute, Cary, NC). All statistical tests were 2‐sided and considered statistically significant if *p* < 0.05.

Participants who completed the dietary questionnaire (Q3) were included in the analyses. We excluded participants with extreme values of the ratio between dietary energy intake and energy requirement (ie, below the 1st [43.6] or above the 99th [195.9] percentiles) and participants not followed after Q3. Patients with prevalent PD at Q3, missing age of diagnosis, and possible PD were also excluded.

To address the potential for reverse causation, we included a 5‐year exposure lag in our main survival analyses. Follow‐up started 5 years after the dietary assessment so that participants censored without PD within the first 5 years of follow‐up or those who developed PD within that period were excluded. This threshold was selected for our main analyses because it is generally considered that loss of dopaminergic neurons in the substantia nigra begins approximately 5 years before diagnosis.^6^

Baseline participants' characteristics were described overall and according to dietary scores categories and PD status at the end of follow‐ up. The frequency of missing values of covariates was small, ranging between 0 and 2%, and similar across categories of dietary scores and PD status. We imputed missing values of covariates with the median for continuous variables and the modal category for categorical variables. Using alternative methods for dealing with missing values (coded as a specific category, multiple imputation) yielded virtually identical results.

Hazards ratios (HRs) and 95% confidence intervals (CI) were estimated using Cox proportional hazards models with age as the time scale. Given the 5‐year–lag, subjects were followed since 5 years after they completed the dietary questionnaire (Q3‐1993) until PD diagnosis or end of follow‐up (maximum of the date of the last questionnaire and last drug reimbursement). The end of the study period was December 31, 2018. The proportional hazards assumption was verified based on Schoenfeld residuals. Two models are presented: the first model was adjusted for age (timescale); and the second was further adjusted for rural residence,^26^ smoking,^26^ parity,^27^ age at menarche,^27^ menopausal status,^27^ physical activity,^28^ caffeine intake,^26^ and total energy intake.^11^, ^12^, ^13^ Continuous covariates (physical activity, caffeine intake, and total energy intake) were modelled as restricted cubic splines with 3 or 4 knots determined based on the lowest Akaike information criterion (AIC) value.

Dietary scores were modeled as continuous or categorical variables based on tertiles of their distribution. We also examined associations for each food component (in tertiles). A *p*‐value for linear trend was estimated by including in the model an ordinal variable defined by the median exposure value in each tertile.

As a secondary objective, we conducted analyses for PD incidence before and after the median age at PD diagnosis (71 years, determined using the Kaplan–Meier method among PD patients). We included a time‐varying covariate in the Cox model indicating if participants were <71 years or ≥71 years old, which allowed us to estimate HRs in both groups and test for differences between them. We examined differences in associations according to age at PD diagnosis for 4 reasons. First, as noted above, a meta‐analysis of the association of the MED diet with PD, prodromal PD, and parkinsonism showed a stronger association for those with diagnosis <60 years,^14^ and because the number of patients with diagnosis <60 years was very small, we used the median age at diagnosis as a cut‐off to compare groups of similar size. Second, there are differences in the PD phenotype according to age at onset,^29^ with evidence of age‐dependent etiologic heterogeneity for some risk factors.^30^, ^31^ Third, survival bias may be an issue for exposures associated with mortality, leading to changes in associations with increasing age.^32^ Fourth, diet was assessed once at baseline and we did not consider changes in diet over the follow‐up. These changes, leading to exposure misclassification, are more likely to be present in older women.

#### Sensitivity Analyses

We repeated the analyses using longer lags of 10, 15, and 20 years. To address the potential for reverse causation, we also performed analyses adjusted for prodromal symptoms (constipation, depression).

We previously showed a positive linear association between plain milk intake and PD, with no association for milk added to coffee and for other dairy products (cheese, yoghurt, and cream).^33^ We generated a modified MED score after exclusion of dairy products and investigated its relationship with PD in analyses that were further adjusted for plain milk intake (continuous variable).

We did not adjust our main analyses for BMI, diabetes, and hypertension because the MED diet is associated with reduced weight gain and lower risk of overweight/obesity, diabetes, and hypertension. These variables may represent mediators of the relation between diet and PD, rather than confounders. Nevertheless, BMI, diabetes, and hypertension collected before Q3 may have influenced diet. We, therefore, repeated the analyses after adjustment for these variables.

Instead of replacing olive oil by ratio of total unsaturated fats to saturated fats for the MED score and berries by polyphenols for the MIND score, we used the original scoring approaches.

We conducted separate analyses for PD incidence before and after 10 years of follow‐up and assessed whether associations were different in the 2 strata using the same approach described above for analyses stratified by median age at diagnosis. In case of non‐differential exposure misclassification, we would expect associations to decrease as follow‐up increases.

### Standard Protocol Approvals, Registrations, and Patient Consents

The study protocol was approved by the French National Commission for Computerized Data and Individual Freedom (Commission Nationale Informatique et Libertés), and all subjects signed informed consent. The protocol is registered at clinicaltrials.gov (NCT03285230).

## Results

### Study Population

Of 74,529 participants who completed the dietary questionnaire, we excluded those with extreme values of energy intake (n = 1,499) or not followed after Q3 (n = 316), as well as prevalent PD patients (n = 40), PD patients with missing age at diagnosis (n = 7), and possible PD patients (n = 36), leaving 72,631 women of whom 908 developed PD (Fig S1). For our main analysis (5‐year–lag), we further excluded women censored without PD within 5 years after Q3 (n = 1,026) and women who developed PD within that period (n = 63). Main analyses are based on 71,542 women followed up to 20 years (mean = 18.8, standard deviation [SD] = 3.3), of whom 845 developed PD (incidence rate = 63.0/100,000 person‐years).

Tables 1A and 1B show baseline participants' characteristics. Mean age was 52.9 years (SD = 6.7). Compared to participants with the lowest scores, those with the highest scores were slightly older, more often postmenopausal, had more children and higher physical activity level, and consumed more caffeine. They were also less likely to be depressed or constipated. Incident PD patients were older, more likely to live in urban areas, more often postmenopausal, more likely to be depressed or constipated, and had more often menarche ≤11 or ≥14 years than other participants (Table S1). They were also less frequently current smokers and less likely to consume caffeine, less often obese, and had lower physical activity levels (Table S1).

**TABLE 1A:** Baseline participant's characteristics overall and according to the scores of adherence to the MED and MIND diets

Characteristics, *N* (%)	Overall *N* = 71,542	Score of adherence to the MED diet		
		Low (0–3) *N* = 20,967 (29.3%)	Medium (4–5) *N* = 32,301 (45.2%)	High (6–9) *N* = 18,274 (25.5%)
Age (yr), M (SD)	52.9 (6.7)	52.6 (6.7)	52.8 (6.6)	53.3 (6.6)
Rural residence	13,474 (18.8)	4123 (19.7)	6073 (18.8)	3278 (17.9)
Menopausal status				
Premenopausal	30,447 (42.6)	9254 (44.1)	13819 (42.8)	7374 (40.4)
Natural menopause	35,230 (49.2)	9987 (47.6)	15898 (49.2)	9345 (51.1)
Artificial menopause	5,402 (7.6)	1587 (7.6)	2372 (7.3)	1443 (7.9)
Unknown type of menopause	463 (0.6)	139 (0.7)	212 (0.7)	112 (0.6)
Parity				
Nulliparous	8,450 (11.8)	2614 (12.5)	3802 (11.8)	2034 (11.1)
One child	11,187 (15.6)	3489 (16.6)	5053 (15.6)	2645 (14.5)
Two children	31,143 (43.5)	8890 (42.4)	14124 (43.7)	8129 (44.5)
≥ Three children	20,762 (29.0)	5974 (28.5)	9322 (28.9)	5466 (29.9)
Age at menarche (yr)				
≤11	14,566 (20.4)	4203 (20.0)	6624 (20.5)	3739 (20.5)
12–13	37,090 (51.8)	10828 (51.6)	16812 (52.0)	9450 (51.7)
≥14	19,886 (27.8)	5936 (28.3)	8865 (27.4)	5085 (27.8)
Smoking				
Never	38,360 (53.6)	11644 (55.5)	17108 (53.0)	9608 (52.6)
Ex	23,531 (32.9)	6460 (30.8)	10769 (33.3)	6302 (34.5)
Current	9,651 (13.5)	2863 (13.7)	4424 (13.7)	2364 (12.9)
Body mass index (kg/m^2^)				
<18.5	2,975 (4.2)	1,034 (4.9)	1,263 (3.9)	678 (3.7)
18.5–24.9	57,284 (80.0)	16,726 (79.8)	25,781 (79.8)	14,777 (80.9)
25.0–29.9	9,508 (13.3)	2,685 (12.8)	4,406 (13.6)	2,417 (13.3)
≥30.0	1,775 (2.5)	522 (2.5)	851 (2.6)	402 (2.2)
Hypertension	26,643 (37.2)	7,880 (37.6)	11,968 (37.1)	6,795 (37.2)
Diabetes	824 (1.2)	230 (1.1)	376 (1.2)	218 (1.2)
Physical activity (MET‐h/week)	49.1 (46.0)	45.9 (45.6)	49.1 (45.7)	52.8 (46.8)
Caffeine intake (mg/day), M (SD)	202.1 (149.7)	195.9 (150.3)	204.2 (149.7)	205.5 (148.7)
Total energy intake (Kcal/day), M (SD)	2210.8 (560.2)	2084.8 (543.6)	2218.1 (565.2)	2342.5 (537.6)
Constipation	17,453 (24.4)	5454 (26.0)	7854 (24.3)	4145 (22.7)
Depression	15,394 (21.5)	4683 (22.3)	6922 (21.4)	3789 (20.7)

Baseline characteristics are shown for participants included in survival analyses with a 5‐year exposure lag.

M = mean; MED = Mediterranean; MET = metabolic equivalent of task; MIND = Mediterranean‐Dietary Approaches to Stop Hypertension Intervention for Neurodegenerative Delay; SD = standard deviation; yr = year.

**TABLE 1B:** Baseline participant's characteristics overall and according to the scores of adherence to the MED and MIND diets

Characteristics, *N* (%)	Overall *N* = 71,542	Score of adherence to the MIND diet		
		Low (<7) *N* = 18,294 (25.6%)	Medium (7‐9) *N* = 31,430 (43.9%)	High (≥9) *N* = 21,818 (30.5%)
Age (yr), M (SD)	52.9 (6.7)	52.4 (6.8)	52.9 (6.7)	53.2 (6.5)
Rural residence	13,474 (18.8)	14820 (81.0)	25448 (81.0)	17800 (81.6)
Menopausal status				
Premenopausal	30,447 (42.6)	8397 (45.9)	13425 (42.7)	8625 (39.5)
Natural menopause	35,230 (49.2)	8392 (45.9)	15459 (49.2)	11379 (52.2)
Artificial menopause	5,402 (7.6)	1383 (7.6)	2359 (7.5)	1660 (7.6)
Unknown type of menopause	463 (0.6)	122 (0.7)	187 (0.6)	154 (0.7)
Parity				
Nulliparous	8,450 (11.8)	2105 (11.5)	3663 (11.7)	2682 (12.3)
One child	11,187 (15.6)	3019 (16.5)	4904 (15.6)	3264 (15.0)
Two children	31,143 (43.5)	8012 (43.8)	13706 (43.6)	9425 (43.2)
≥ Three children	20,762 (29.0)	5158 (28.2)	9157 (29.1)	6447 (29.5)
Age at menarche (yr)				
≤11	14,566 (20.4)	3463 (18.9)	6324 (20.1)	4779 (21.9)
12–13	37,090 (51.8)	9455 (51.7)	16297 (51.9)	11338 (52.0)
≥14	19,886 (27.8)	5376 (29.4)	8809 (28.0)	5701 (26.1)
Smoking				
Never	38,360 (53.6)	10660 (58.3)	17071 (54.3)	10629 (48.7)
Ex	23,531 (32.9)	5264 (28.8)	10199 (32.4)	8068 (37.0)
Current	9,651 (13.5)	2370 (13.0)	4160 (13.2)	3121 (14.3)
Body mass index (kg/m^2^)				
<18.5	2,975 (4.2)	985 (5.5)	1,293 (4.2)	697 (3.3)
18.5–24.9	57,284 (80.0)	14,021 (78.4)	24,548 (79.7)	17,192 (80.6)
25.0–29.9	9,508 (13.3)	2,401 (13.4)	4,113 (13.4)	2,994 (14.0)
≥30.0	1,775 (2.5)	473 (2.7)	846 (2.8)	456 (2.2)
Hypertension	26,643 (37.2)	6,981 (38.2)	11,674 (37.1)	7,988 (36.6)
Diabetes	824 (1.2)	193 (1.0)	358 (1.1)	273 (1.3)
Physical activity (MET‐h/week)	49.1 (46.0)	44.9 (42.7)	48.8 (46.0)	53.0 (48.3)
Caffeine intake (mg/day), M (SD)	202.1 (149.7)	174.2 (138.4)	200.7 (148.7)	227.4 (155.8)
Total energy intake (Kcal/day), M (SD)	2210.8 (560.2)	2211.2 (572.9)	2219.0 (563.2)	2198.8 (544.6)
Constipation	17,453 (24.4)	4520 (24.7)	7701 (24.5)	5232 (24.0)
Depression	15,394 (21.5)	4166 (22.8)	6560 (20.9)	4668 (21.4)

Baseline characteristics are shown for participants included in survival analyses with a 5‐year exposure lag.

M = mean; MED = Mediterranean; MET = metabolic equivalent of task; MIND = Mediterranean‐Dietary Approaches to Stop Hypertension Intervention for Neurodegenerative Delay; SD = standard deviation; yr = year.

### Dietary Scores and PD Incidence

Tables 2A and 2B shows the association between dietary scores and PD with a 5‐year–lag. Overall, there was no association between the MED (HR per 1‐unit = 0.98, 95% CI = 0.94–1.03) or MIND (HR per 1‐unit = 1.00, 95% CI = 0.96–1.04) diets with PD.

**TABLE 2A:** Association between the scores of adherence to the MED and MIND diet and PD incidence

MED diet	*N* cases (IR/100,000)	Model 1			Model 2		
		HR (95% CI)	*p*‐trend	*p*‐int.^a^	HR (95% CI)	*p*‐trend	*p*‐int.^a^
Total population							
Low	236 (60.1)	1.00 (Reference)			1.00 (Reference)		
Medium	388 (64.0)	1.04 (0.89‐1.23)			1.02 (0.87‐1.20)		
High	221 (64.4)	1.01 (0.84‐1.22)			0.97 (0.80‐1.17)		
Per 1‐unit	845 (63.0)	1.00 (0.96‐1.04)	0.845		0.98 (0.94‐1.03)	0.482	
Low/medium	624 (62.5)	1.00 (Reference)			1.00 (Reference)		
High	221 (64.4)	0.99 (0.85‐1.15)			0.96 (0.82‐1.12)		
PD diagnosis < 71 yr							
Low	97 (35.8)	1.00 (Reference)			1.00 (Reference)		
Medium	155 (37.6)	1.04 (0.81‐1.34)			1.00 (0.77‐1.29)		
High	69 (30.6)	0.83 (0.61‐1.13)			0.76 (0.56‐1.04)		
Per 1‐unit	321 (35.3)	0.95 (0.88‐1.01)	0.106		**0.93 (0.86‐0.99)**	**0.029**	
Low/medium	252 (36.9)	1.00 (Reference)			1.00 (Reference)		
High	69 (30.6)	0.81 (0.62‐1.06)			**0.76 (0.58‐1.00)**		
PD diagnosis ≥ 71 yr							
Low	139 (113.0)	1.00 (Reference)			1.00 (Reference)		
Medium	233 (118.6)	1.05 (0.85‐1.29)			1.04 (0.84‐1.29)		
High	152 (128.6)	1.13 (0.90‐1.43)			1.11 (0.88‐1.41)		
Per 1‐unit	524 (119.7)	1.03 (0.97‐1.08)	0.309	0.058	1.02 (0.97‐1.08)	0.408	**0.026**
Low/medium	372 (116.4)	1.00 (Reference)			1.00 (Reference)		
High	152 (128.6)	1.10 (0.91‐1.33)		0.065	1.08 (0.90‐1.31)		**0.038**

HR and 95% CI calculated using Cox proportional hazards models with age as the time scale. Statistically significant HRs and *p*‐values are bolded. Model 1: adjusted for age (as the timescale). Model 2: further adjusted for baseline place of residence (rural/urban), age at menarche (≤11/12–13/≥14 yr), parity (nulliparous/1 child/2 children/≥3 children), smoking (never/ex/current), menopausal status (premenopausal/natural/artificial/unknown type), physical activity (RCS‐4 knots), caffeine intake (RCS‐4 knots), and total energy intake (RCS‐4 knots).

CI = confidence interval; HR = hazard ratio; int. = interaction; IR = crude incidence rate per 100,000 person‐yr; MED = Mediterranean; MIND = Mediterranean‐Dietary Approaches to Stop Hypertension Intervention for Neurodegenerative Delay; PD = Parkinson's disease; RCS = restricted cubic spline; yr = year.

^a^ *P*‐value for the difference in HRs between the two age strata.

**TABLE 2B:** Association between the scores of adherence to the MED and MIND diet and PD incidence

MIND diet	*N* cases (IR/100,000)	Model 1			Model 2		
		HR (95% CI)	*p*‐trend	*p*‐int.^a^	HR (95% CI)	*p*‐trend	*p*‐int.^a^
Total population							
Low	200 (58.4)	1.00 (Reference)			1.00 (Reference)		
Medium	390 (67.0)	1.09 (0.92‐1.29)			1.07 (0.90‐1.27)		
High	255 (66.1)	1.00 (0.83‐1.20)			0.98 (0.81‐1.18)		
Per 1‐unit	845 (62.3)	1.00 (0.96‐1.04)	0.949		1.00 (0.96‐1.04)	0.916	
Low/medium	590 (63.3)	1.00 (Reference)			1.00 (Reference)		
High	255 (62.3)	0.94 (0.82‐1.09)			0.93 (0.81‐1.08)		
PD diagnosis < 71 yr							
Low	82 (34.3)	1.00 (Reference)			1.00 (Reference)		
Medium	158 (39.7)	1.13 (0.87‐1.48)			1.11 (0.85‐1.45)		
High	81 (29.9)	0.84 (0.61‐1.14)			0.81 (0.59‐1.10)		
Per 1‐unit	321 (35.3)	0.97 (0.91‐1.04)	0.397		0.96 (0.90‐1.03)	0.292	
Low/medium	240 (37.6)	1.00 (Reference)			1.00 (Reference)		
High	81 (29.9)	**0.77 (0.60‐0.99)**			**0.75 (0.58‐0.97)**		
PD diagnosis ≥ 71 yr							
Low	118 (113.0)	1.00 (Reference)			1.00 (Reference)		
Medium	232 (120.0)	1.06 (0.85‐1.32)			1.06 (0.85‐1.32)		
High	174 (124.3)	1.10 (0.87‐1.38)			1.10 (0.87‐1.39)		
Per 1‐unit	524 (119.7)	1.02 (0.97‐1.08)	0.446	0.255	1.02 (0.97‐1.08)	0.447	0.194
Low/medium	350 (117.6)	1.00 (Reference)			1.00 (Reference)		
High	174 (124.3)	1.06 (0.88‐1.27)		**0.045**	1.06 (0.88‐1.27)		**0.035**

HR and 95% CI calculated using Cox proportional hazards models with age as the time scale. Statistically significant HRs and *p*‐values are bolded. Model 1: adjusted for age (as the timescale). Model 2: further adjusted for baseline place of residence (rural/urban), age at menarche (≤11/12–13/≥14 yr), parity (nulliparous/1 child/2 children/≥3 children), smoking (never/ex/current), menopausal status (premenopausal/natural/artificial/unknown type), physical activity (RCS‐4 knots), caffeine intake (RCS‐4 knots), and total energy intake (RCS‐4 knots).

CI = confidence interval; HR = hazard ratio; int. = interaction; IR = crude incidence rate per 100,000 person‐yr; MED = Mediterranean; MIND = Mediterranean‐Dietary Approaches to Stop Hypertension Intervention for Neurodegenerative Delay; PD = Parkinson's disease; RCS = restricted cubic spline; yr = year.

^a^ *P*‐value for the difference in HRs between the two age strata.

The relation between the MED diet and PD was significantly different according to median age at PD diagnosis. There was a significant linear trend of decreasing PD incidence with increasing MED scores for those who developed PD <71 years (HR/1‐unit = 0.93, 95% CI = 0.86–0.99, *p* = 0.029; *p*‐Age × MED interaction = 0.026). Participants with high adherence tended to have lower PD incidence <71 years than those with low adherence (HR = 0.76, 95% CI = 0.56–1.04) with no difference for the medium versus low category (HR = 1.00, 95% CI = 0.77–1.29). Compared to the low + medium groups combined, participants with high adherence had 23% lower PD incidence <71 years (HR = 0.76, 95% CI = 0.58–1.00; *p*‐Age × MED interaction = 0.038). There was no association between the MED diet and PD incidence ≥71 years.

We observed a similar pattern for the MIND diet. Participants in the high group had 25% lower PD incidence <71 years compared to those in the low + medium groups (HR = 0.75, 95% CI = 0.58–0.97; *p*‐Age × MIND interaction = 0.035) without a significant trend (HR/1‐unit = 0.96, 95% CI = 0.90–1.03, *p* = 0.292; *p*‐Age × MIND interaction = 0.194). There was no association with PD incidence ≥71 years.

### Individual Components of Dietary Scores and PD Incidence

Table 3 shows associations of each component of the MED score with PD. Table S2 shows the frequency of consumption of individual components. We found an inverse association between legumes and PD, both overall (HR_T3vsT1_ = 0.79, 95% CI = 0.64–0.97, *p*‐trend = 0.027) and <71 years (HR_T3vsT1_ = 0.64, 95% CI = 0.46–0.88, *p*‐trend = 0.012). An inverse association was also observed for PD incidence <71 years for high unsaturated fat/saturated fat ratio (HR_T3vsT1_ = 0.70, 95% CI = 0.50–0.99, *p*‐trend = 0.047). Other components were not significantly associated with PD, but the highest intake groups of all other components were characterized by HRs <1 for PD incidence <71 years, except for meat, dairy, and alcohol.

**TABLE 3:** Association between the intake of dietary components (in tertiles) of the MED score and PD incidence

MED score component	Overall		PD diagnosis < 71 years		PD diagnosis ≥ 71 years	
	HR (95% CI)	*p*‐Trend	HR (95% CI)	*p*‐Trend	HR (95% CI)	*p*‐Trend
Vegetables (g/day)						
<182.4	1.00 (Reference)		1.00 (Reference)		1.00 (Reference)	
182.4–349.9	0.94 (0.79‐1.11)		0.79 (0.61‐1.04)		1.05 (0.84‐1.31)	
≥349.9	0.97 (0.80‐1.18)	0.867	0.84 (0.62‐1.14)	0.345	1.08 (0.84‐1.39)	0.560
Legumes (g/day)						
<3.4	1.00 (Reference)		1.00 (Reference)		1.00 (Reference)	
3.4–28.6	0.89 (0.79‐1.08)		0.75 (0.58‐0.97)		0.99 (0.80‐1.21)	
≥28.6	**0.79 (0.64‐0.97)**	**0.027**	**0.64 (0.46‐0.88)**	**0.012**	0.91 (0.70‐1.18)	0.445
Fruits (g/day)						
<138.6	1.00 (Reference)		1.00 (Reference)		1.00 (Reference)	
138.6–330.7	0.89 (0.75‐1.06)		0.85 (0.65‐1.10)		0.93 (0.74‐1.17)	
≥330.7	1.01 (0.83‐1.22)	0.688	0.85 (0.62‐1.16)	0.332	1.12 (0.87‐1.43)	0.204
Cereals (g/day)						
<119.1	1.00 (Reference)		1.00 (Reference)		1.00 (Reference)	
119.1–246.6	1.01 (0.83‐1.22)		1.03 (0.77‐1.39)		0.99 (0.79‐1.23)	
≥246.6	0.90 (0.72‐1.33)	0.340	0.83 (0.57‐1.21)	0.259	0.95 (0.71‐1.27)	0.736
Fish (g/day)						
<12.4	1.00 (Reference)		1.00 (Reference)		1.00 (Reference)	
12.4–37.3	1.07 (0.88‐1.31)		0.95 (0.70‐1.28)		1.17 (0.90‐1.51)	
≥37.3	1.02 (0.81‐1.28)	0.905	0.90 (0.62‐1.31)	0.608	1.11 (0.82‐1.49)	0.793
Meat (g/day)						
<67.9	1.00 (0.82‐1.24)		1.01 (0.73‐1.40)		1.02 (0.78‐1.33)	
67.9–132.2	1.04 (0.87‐1.23)		0.95 (0.73‐1.24)		1.11 (0.88‐1.40)	
≥132.2	1.00 (Reference)	0.950	1.00 (Reference)	0.984	1.00 (Reference)	0.926
Dairy product (g/day)						
<117.9	1.03 (0.85‐1.24)		0.94 (0.70‐1.28)		1.09 (0.85‐1.39)	
117.9–342.2	0.99 (0.83‐1.16)		0.84 (0.64‐1.09)		1.09 (0.88‐1.35)	
≥342.2	1.00 (Reference)	0.850	1.00 (Reference)	0.532	1.00 (Reference)	0.472
Unsaturated fat:saturated fat ratio						
<1.1	1.00 (Reference)		1.00 (Reference)		1.00 (Reference)	
1.1–1.5	0.89 (0.76‐1.04)		0.90 (0.70‐1.16)		0.88 (0.72‐1.09)	
≥1.5	0.88 (0.72‐1.07)	0.193	**0.70 (0.50‐0.99)**	**0.047**	0.98 (0.77‐1.26)	0.923
Alcohol (g/day)						
<5	0.99 (0.86‐1.15)		0.97 (0.76‐1.24)		1.01 (0.83‐1.21)	
5–25	1.00 (Reference)		1.00 (Reference)		1.00 (Reference)	
≥25	1.03 (0.83‐1.27)	0.765	1.13 (0.81‐1.57)	0.400	0.96 (0.73‐1.27)	0.771

HR and 95% CI calculated using Cox proportional hazards models with age as the time scale; RCS, restricted cubic spline. Statistically significant HRs and *p*‐values are bolded. Model 1: adjusted for age (as the timescale). The lowest category is the reference except for meat, dairy, and alcohol. Model 2: further adjusted for baseline place of residence (rural/urban), age at menarche (≤11/12–13/≥14 years), parity (nulliparous/1 child/2 children/≥3 children), smoking (never/ex/current), menopausal status (premenopausal/natural/artificial/unknown type), physical activity (RCS‐4 knots), caffeine intake (RCS‐4 knots) and total energy intake (RCS‐4 knots).

CI = confidence interval; HR = hazard ratio; MED = Mediterranean; MIND = Mediterranean‐Dietary Approaches to Stop Hypertension Intervention for Neurodegenerative Delay; PD = Parkinson's disease; RCS = restricted cubic spline.

**TABLE 4A:** Association between the intake of dietary components of the MIND score and PD incidence

MIND score component	Overall		PD diagnosis < 71 years		PD diagnosis ≥ 71 years	
	HR (95% CI)	*pp*‐Trend	HR (95% CI)	*p*‐Trend	HR (95% CI)	*p*‐Trend
Whole grains						
0 g/day	1.00 (Reference)		1.00 (Reference)		1.00 (Reference)	
>0 g/day	1.08 (0.94‐1.24)		1.14 (0.91‐1.42)		1.05 (0.88‐1.25)	
Nuts						
0 g/day	1.00 (Reference)		1.00 (Reference)		1.00 (Reference)	
>0 g/day	0.95 (0.82‐1.10)		1.01 (0.78‐1.31)		0.93 (0.77‐1.11)	
Total polyphenols						
<1186 mg/day	1.00 (Reference)		1.00 (Reference)		1.00 (Reference)	
1186‐1888 mg/day	1.04 (0.85‐1.28)		1.19 (0.84‐1.67)		0.97 (0.75‐1.26)	
≥1888 mg/day	1.11 (0.82‐1.50)	0.499	1.19 (0.74‐1.94)	0.532	1.09 (0.74‐1.59)	0.660
Green leafy vegetables						
<73 g/day	1.00 (Reference)		1.00 (Reference)		1.00 (Reference)	
73‐131 g/day	1.00 (0.85‐1.17)		0.92 (0.71‐1.18)		1.06 (0.86‐1.30)	
≥131 g/day	1.07 (0.89‐1.28)	0.461	0.95 (0.70‐1.28)	0.714	1.16 (0.92‐1.47)	0.205
Olive oil						
<median (3.3)	1.00 (Reference)		1.00 (Reference)		1.00 (Reference)	
≥ median (3.3)	0.95 (0.83‐1.09)		0.81 (0.65‐1.01)		1.06 (0.89‐1.26)	
Other vegetables						
<7 times/week	1.00 (Reference)		1.00 (Reference)		1.00 (Reference)	
7‐14 times/week	0.92 (0.53‐1.61)		0.97 (0.38‐2.44)		0.89 (0.44‐1.79)	
>14 times/week	0.91 (0.53‐1.56)	0.783	0.94 (0.39‐2.31)	0.852	0.88 (0.45‐1.72)	0.805
Beans						
<1 time/month	1.00 (Reference)		1.00 (Reference)		1.00 (Reference)	
1/month‐1 time/week	0.94 (0.81‐1.10)		0.82 (0.64‐1.04)		1.03 (0.86‐1.25)	
≥2 times/week	0.84 (0.66‐1.06)	0.146	**0.66 (0.45‐0.96)**	**0.016**	0.99 (0.73‐1.34)	0.910
Fish						
<1 time/week	1.00 (Reference)		1.00 (Reference)		1.00 (Reference)	
1 time/week	1.17 (0.93‐1.49)		1.02 (0.71‐1.48)		1.28 (0.94‐1.74)	
≥2 times/week	1.12 (0.88‐1.42)	0.717	0.96 (0.66‐1.41)	0.706	1.23 (0.90‐1.68)	0.446

HR and 95% CI calculated using Cox proportional hazards models with age as the time scale. Statistically significant HRs and *p*‐values are bolded. Models (Models 2) are adjusted for age (as the timescale), baseline place of residence (rural/urban), age at menarche (≤11/12‐13/≥14 years), parity (nulliparous/one child/two children/≥three children), smoking (never/ex/current), menopausal status (premenopausal/natural/artificial/unknown type), physical activity (RCS‐4 knots), caffeine intake (RCS‐4 knots) and total energy intake (RCS‐4 knots). Categories are based on tertiles, except for whole grains, nuts, and olive oil.

CI = confidence interval; HR = hazard ratio; MED = Mediterranean; MIND = Mediterranean‐Dietary Approaches to Stop Hypertension Intervention for Neurodegenerative Delay; PD = Parkinson's disease; RCS = restricted cubic spline.

**TABLE 4B:** Association between the intake of dietary components of the MIND score and PD incidence

MIND score component	Overall		PD diagnosis < 71 years		PD diagnosis ≥ 71 years	
	HR (95% CI)	*p*‐Trend	HR (95% CI)	*P*‐Trend	HR (95% CI)	*p*‐Trend
Poultry						
<1 time/week	1.00 (Reference)		1.00 (Reference)		1.00 (Reference)	
1 time/week	0.96 (0.83‐1.11)		0.89 (0.70‐1.13)		1.00 (0.83‐1.20)	
≥2 times/week	0.72 (0.52‐1.00)	0.118	0.61 (0.37‐1.03)	0.080	0.80 (0.52‐1.23)	0.543
Red meats & products						
≥7 meals/week	1.00 (Reference)		1.00 (Reference)		1.00 (Reference)	
5–6 meals/week	1.00 (0.80‐1.25)		0.81 (0.59‐1.11)		1.23 (0.90‐1.69)	
≤4 meals/week	1.05 (0.83‐1.32)	0.611	0.90 (0.64‐1.26)	0.739	1.23 (0.89‐1.71)	0.349
Cheese						
>7 times/week	1.00 (Reference)		1.00 (Reference)		1.00 (Reference)	
1–7 times/week	0.96 (0.82‐1.12)		1.00 (0.78‐1.29)		0.93 (0.77‐1.13)	
<1 time/week	1.22 (0.90‐1.66)	0.850	0.93 (0.52‐1.68)	0.931	1.37 (0.96‐1.96)	0.758
Pastries & sweets						
≥7 times/week	1.00 (Reference)		1.00 (Reference)		1.00 (Reference)	
5–6 times/week	0.90 (0.72‐1.14)		0.97 (0.67‐1.41)		0.86 (0.64‐1.16)	
<5 times/week	1.00 (0.86‐1.16)	0.823	1.16 (0.90‐1.49)	0.311	0.92 (0.75‐1.11)	0.299
Fried & fast food						
>2 times/week	1.00 (Reference)		1.00 (Reference)		1.00 (Reference)	
1–2 times/week	0.90 (0.45‐1.84)		0.68 (0.28‐1.68)		1.28 (0.41‐4.03)	
<1 time/week	1.04 (0.52‐2.11)	0.124	0.86 (0.35‐2.11)	0.181	1.38 (0.44‐4.32)	0.377
Butter & margarine						
>14 times/week	1.00 (Reference)		1.00 (Reference)		1.00 (Reference)	
7–14 times/week	0.85 (0.69‐1.05)		0.95 (0.69‐1.33)		0.78 (0.59‐1.03)	
<7 times/week	0.89 (0.72‐1.09)	0.404	0.86 (0.62‐1.18)	0.295	0.90 (0.69‐1.17)	0.828
Wine						
Never or >10 glasses/week	1.00 (Reference)		1.00 (Reference)		1.00 (Reference)	
1/month‐6 glasses/week	1.05 (0.91‐1.21)		1.02 (0.81‐1.29)		1.07 (0.89‐1.28)	
7–10 glasses/week	0.94 (0.74‐1.19)	0.668	0.78 (0.52‐1.18)	0.267	1.04 (0.78‐1.39)	0.751

HR and 95% CI calculated using Cox proportional hazards models with age as the time scale. Statistically significant HRs and *p*‐values are bolded. Models (Models 2) are adjusted for age (as the timescale), baseline place of residence (rural/urban), age at menarche (≤11/12‐13/≥14 years), parity (nulliparous/one child/two children/≥three children), smoking (never/ex/current), menopausal status (premenopausal/natural/artificial/unknown type), physical activity (RCS‐4 knots), caffeine intake (RCS‐4 knots) and total energy intake (RCS‐4 knots). Categories are based on tertiles.

CI = confidence interval; HR = hazard ratio; MED = Mediterranean; MIND = Mediterranean‐Dietary Approaches to Stop Hypertension Intervention for Neurodegenerative Delay; PD = Parkinson's disease; RCS = restricted cubic spline.

In analyses for components of the MIND diet, higher beans consumption (HR = 0.66, 95% CI = 0.45–0.96, *p*‐trend = 0.016) was associated with lower PD incidence <71 years (Tables 4A and 4B, Table S3). Olive oil intake also tended to be associated with lower PD incidence albeit not significantly. Other components were not associated with PD.

### Sensitivity Analyses

**FIGURE 1:**
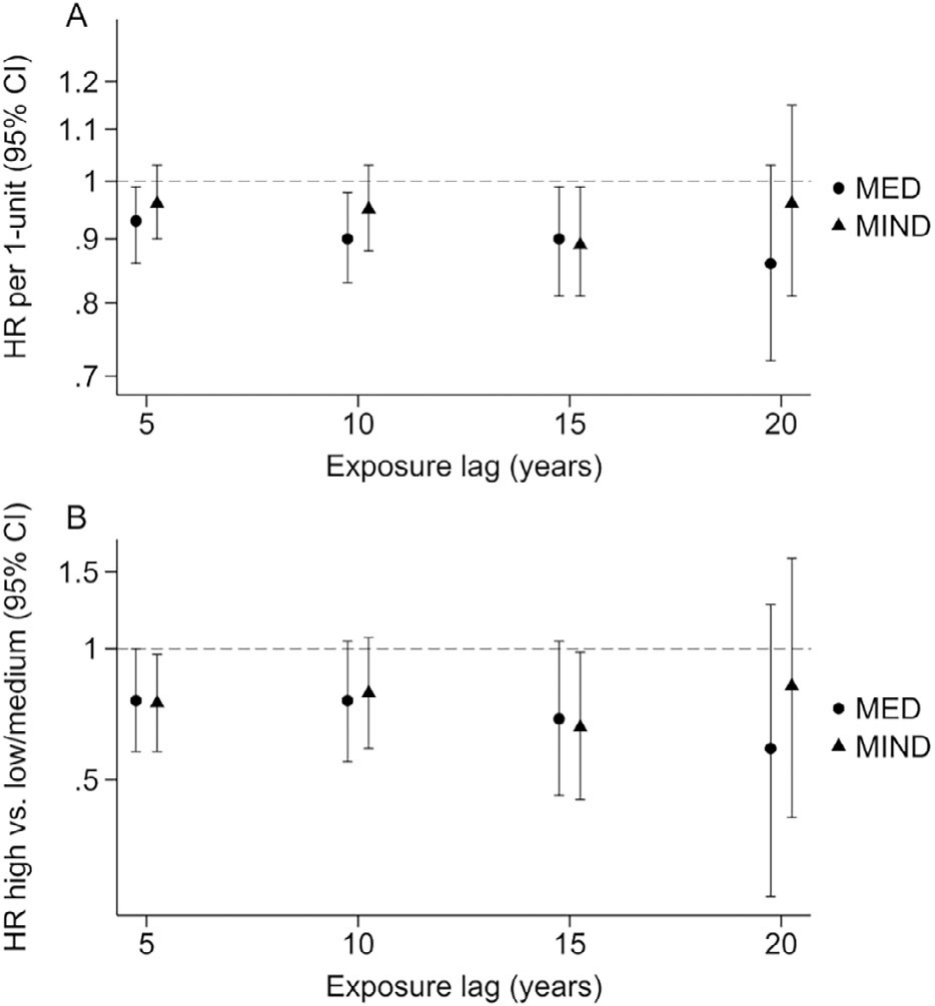
Association between scores of adherence to the Mediterranean (MED) (circles) and Mediterranean‐Dietary Approaches to Stop Hypertension Intervention for Neurodegenerative Delay (MIND) (triangles) diets and Parkinson's disease (PD) incidence before 71 years for exposure lags of 5, 10, 15, and 20 years. Hazard ratios (HR) and 95% confidence intervals (CI) from Cox proportional hazards models with age as the time scale adjusted for baseline place of residence (rural/urban), age at menarche (≤11/12–13/≥14 years), parity (nulliparous/1 child/2 children/≥3 children), smoking (never/ex/current), menopausal status (premenopausal/natural/artificial/unknown type), physical activity (restricted cubic spline [RCS]‐4 knots), caffeine intake (RCS‐4 knots), and total energy intake (RCS‐4 knots). A shows HRs for trend (/1‐unit), and B, shows HRs comparing participants in the high category to those in the low and medium categories combined.

The number of participants available for survival analyses decreased with increasing lags (Fig 1 and Fig S1, Tables S4–S6). Compared to main analyses, trends of decreasing PD incidence <71 years with increasing adherence to the MED diet were similar for a 10‐year–lag (HR = 0.90, 95% CI = 0.83–0.98, *p* = 0.014) and 15‐year–lag (HR = 0.90, 95% CI = 0.81–0.99, *p* = 0.038). The association was slightly stronger for a 20‐year–lag, but not statistically significant because of a smaller number of events (HR = 0.86, 95% CI = 0.72–1.03, *p* = 0.102).

For the MIND diet, the reduction in PD incidence <71 years in participants in the top tertile compared to those in the bottom + middle tertiles was similar for a 10‐year–lag compared to main analyses, but not statistically significant (HR = 0.79, 95% CI = 0.59–1.06). This inverse association became stronger for a 15‐year–lag (HR = 0.66, 95% CI = 0.45–0.98) with a significant trend (*p*‐trend = 0.025). Analyses with a 20‐year–lag yielded similar findings, but associations were not statistically significant because of a smaller number of events.

Analyses adjusted for prodromal symptoms (Table S7), based on a modified MED score after exclusion of dairy products (Table S8), adjusted for BMI, diabetes, and hypertension (Table S9) or using the original scoring approaches of dietary scores (Table S10) led to similar conclusions as main analyses.

In analyses stratified by duration of follow‐up, associations between dietary scores and PD incidence were not statistically different over the first 10 years of follow‐up and after, overall (MED, *p*‐interaction = 0.339; MIND, *p*‐interaction = 0.418) and <71 years (MED, *p*‐interaction = 0.487; MIND, *p*‐interaction = 0.381).

## Discussion

In this large prospective cohort of women with a long follow‐up, there was no association between adherence to the MED or MIND diets and PD incidence overall. However, higher adherence to both diets was associated with lower PD incidence <71 years, but not with later incidence.

Our findings extend those from 3 previous cohort studies.^11^, ^12^, ^13^ In 2 US cohorts (318 male + 190 female PD cases), participants in the top quintile of adherence to the MED diet had lower PD risk compared to those in the bottom quintile (HR = 0.75, 95% CI = 0.57–1.00; *p*‐trend = 0.07). This association was present in women (HR = 0.66, 95% CI = 0.43–1.00; *p*‐trend = 0.09) and analyses with a 4‐year–lag (HR not available).^11^ In a Swedish cohort of women (101 PD cases), greater adherence to the MED diet in middle‐age was associated with lower PD risk (HR high vs low = 0.54, 95% CI = 0.0.30–0.98; *p*‐trend = 0.049). Contrary to our findings, the association was stronger for women who developed PD ≥65 years (n = 31) than earlier (n = 70), but comparisons are difficult because of differences in the age distribution and lengths of follow‐up between the 2 studies.^13^ In the Rotterdam study (68 male + 61 female PD cases), the MED diet was associated with lower PD risk, although not significantly (overall, HR/1‐SD = 0.80, 95% CI = 0.50–1.29; women, HR/1‐SD = 0.93, 95% CI = 0.69–1.25).^12^ Inconsistent results may be explained by differences in study design and/or size, PD definition, populations, dietary assessment, and residual confounding because not all studies adjusted on behaviors (eg, caffeine, physical activity). Two other studies showed that higher adherence to the MED diet were associated with reduced risk of prodromal symptoms (constipation, rapid eye movement sleep behavior disorder, hyposmia, excessive daytime sleepiness, and depressive symptoms).^34^, ^35^

Regarding the MIND diet, 1 study (706 older adults of whom 302 developed parkinsonism, follow‐up = 4 years) showed that it was associated with decreased risk and slower progression of parkinsonism, and each unit increase was associated with a 13% reduction in parkinsonism incidence.^36^ Parkinsonism can be caused by several conditions (stroke, drugs, and neuro‐degenerative diseases, including PD). One study of 167 prevalent PD patients (average disease duration = 6.5 years) and 119 controls assessed diet over the past year.^37^ MIND scores were similar in patients and controls. Among patients, higher scores were associated with later onset, especially in women. The cross‐sectional study design and the dietary assessment after disease onset strongly limit the ability to compare results from this study and our own. There are no prospective studies on the relation between the MIND diet and PD.

One of the main differences between our study and previous ones^14^ lies in our ability to identify a considerably larger number of women who developed PD over a longer follow‐up. We included in our analyses exposure lags of up to 20 years and adjusted for prodromal symptoms to address the potential for reverse causation. Consistent findings of analyses with a 5‐year–lag and those using longer lags or adjusted for prodromal symptoms suggest that the inverse association of adherence to the MED/MIND diets and PD incidence <71 years is unlikely to be explained by reverse causation.

We found a significant difference in the association between dietary scores and PD according to age at PD diagnosis. Previous studies also reported that associations between the MED score and other outcomes (eg, frailty,^38^ mortality^39^) decrease as age increases. A meta‐analysis of the association of the MED diet with PD, prodromal PD, and parkinsonism, reported that the association was stronger for those with younger onset.^14^ Possible explanations for age‐related differences include age‐dependent etiologic heterogeneity and selective survival.^32^ More pronounced exposure measurement error in older participants could also contribute given that diet was assessed at baseline only and changes in diet are likely to be more important at older ages. However, this hypothesis seems less likely as analyses stratified by length of follow‐up in those with PD onset ≥71 years did not show marked differences in the 2 groups.

The MED/MIND diets are characterized by high intake of fruits, vegetables, and olive oil, all of which are rich sources of nutrients and bioactive compounds (eg, vitamin E, n‐3 fatty acids, flavonoids, vitamin A, and carotenoids) with antioxidant and anti‐inflammatory properties that have been shown to have beneficial effects for brain health and outcomes such as Alzheimer disease and cognitive decline.^40^ Various pathways are hypothesized to explain the relationship between diet and PD, including the modulation of the gut microbiome, oxidative stress, and immune and inflammatory processes.^41^

In our study, legumes and a high ratio of unsaturated to saturated fat had the strongest contribution to the inverse association between the MED diet and PD incidence <71 years. Other components may have subtle individual impacts that are discernible only as part of an overall dietary pattern, particularly when cumulative and synergistic effects are taken into account.^9^ Except for meat, dairy, and alcohol, the highest intake groups of all other components of the MED diet were characterized by HRs <1 for PD incidence <71 years. Legumes are optimal sources of phenolic compounds and anthocyanins, which can reduce oxidative stress and inflammation.^42^ A recent study found an inverse association between legumes consumption and prodromal PD probability in patients age <70 years.^43^ Unsaturated fatty acids, especially polyunsaturated fatty acids, are crucial components in the membranes of neurons and glial cells, and regulate pro/anti‐inflammatory cytokines that could play a role in neurodegenerative diseases.^44^ According to a recent meta‐analysis, a higher intake of unsaturated fatty acids is associated with reduced PD risk.^45^

Beans and olive oil had the strongest contribution to the association of the MIND diet with PD incidence <71 years. Beans are part of the legume group from the MED diet that was inversely associated with PD. Several types of beans contain levodopa, and there are anecdotal reports of improvement in motor symptoms in PD patients after administration of beans.^46^ There was also an inverse association between olive oil intake, a rich source of monounsaturated fatty acids, and PD, in agreement with findings for the MED diet.

The prospective design, the large sample size, and long follow‐up represent the main strengths of our study. The prospective design ensured that data collection before PD onset was not subject to recall bias. Moreover, the long follow‐up allowed us to study long‐term associations between dietary scores and PD by performing lagged analyses to address reverse causation. Compared to previous studies, our study is based on a considerably larger number of PD patients. Finally, our analyses are adjusted for a wide range of potential confounders.

However, some limitations need to be acknowledged. First, diet was only assessed at baseline, and exposure misclassification is possible, as dietary habits may change over time. Given the prospective design, misclassification is likely to be non‐differential and attenuate true associations. Studies that compared analyses based on dietary scores assessed at baseline or updated during the follow‐up showed no important difference in associations or a small attenuation for analyses using the baseline measure only,^47^, ^48^ possibly because dietary behaviors, in particular adherence to the MED diet, are relatively stable.^49^ We addressed this issue by conducting analyses stratified by duration of follow‐up and found no marked differences before and after 10 years of follow‐up. Second, although we used a validated dietary questionnaire, self‐reported diet could result in non‐differential exposure misclassification. Third, we replaced berry intake by total polyphenol intake for the MIND diet, and although Phenol‐Explorer is the most comprehensive food composition database on polyphenols, it has incomplete coverage, assumes uniform content, and does account for bioavailability. Hence, measurement error in dietary polyphenol intake may have led to underestimate associations. Fourth, despite the adjustment for several confounders, we cannot rule out unmeasured confounding. Fifth, E3N participants are mostly teachers, with relatively healthier lifestyles and higher education than the general population, which limits generalizability. However, there are several examples of associations in occupational cohorts that are similar to those in the general population, and some authors argue that representativeness is not crucial for estimating associations.^50^ In addition, PD incidence rates in E3N are in agreement with those in women from Western Europe,^16^ and selection into the study did not result in lower rates. Finally, our study population was composed of women, which hampers generalizability to men, nevertheless, women are an under‐represented population in PD research, warranting further studies.^51^

In conclusion, in women, the MED and MIND diets were associated with lower incidence of PD <71 years. Promoting a healthy diet might help prevent or delay PD.^1^ Further studies are warranted to confirm our findings, to understand the effects of individual components of dietary patterns, and to elucidate underlying mechanisms.

## Author Contributions

M.H., F.R.M., and A.E. contributed to the conception and design of the study; M.H., E.C., F.A., E.R., F.R.M., and A.E. contributed to the acquisition and analysis of data; all authors contributed to contributed to drafting the text or preparing the figures.

## Potential Conflicts of Interest

Nothing to report.

## Supporting information


**Figure S1.** Flow chart for inclusion into the study (5, 10, 15, and 20 years‐lags).
**Figure S2**. Directed acyclic graph.
**Table S1**. Participant's characteristics at baseline (1993‐Q3) according to Parkinson's disease status at the end of the follow‐up.
**Table S2**. Frequency of consumption of individual components of the Mediterranean (MED) diet score.
**Table S3**. Frequency of consumption of individuals components of the MIND diet score.
**Table S4**. Association between the scores of adherence to the Mediterranean (MED) and MIND diet and PD incidence: analyses lagged by 10 years.
**Table S5**. Association between the scores of adherence to the Mediterranean (MED) and MIND diet and PD incidence: analyses lagged by 15 years.
**Table S6**. Association between the scores of adherence to the Mediterranean (MED) and MIND diet and PD incidence: analyses lagged by 20 years.
**Table S7**. Association between the scores of adherence to the Mediterranean (MED) and MIND diet and PD incidence: analyses adjusted for constipation and depression.
**Table S8**. Association between the scores of adherence to the modified Mediterranean (MED) diet (after exclusion of dairy products) and PD incidence.
**Table S9**. Association between the scores of adherence to the Mediterranean (MED) and MIND diet and PD incidence: analyses adjusted for BMI, diabetes, and hypertension.
**Table S10**. Association between the scores of adherence to the Mediterranean (MED) and MIND diet and PD incidence: analyses using the original scoring system for the MED and MIND diet scores.

## Data Availability

Data on E3N cohort participants are available to bona fide researchers for all type of health‐related research, which is in the public interest. Data are made available under managed access owing to governance constraints and need to protect the privacy of study participants. Raw data requests should be submitted through the E3N website (www.e3n.fr) or sent to contact@e3n.fr; requests will be reviewed by the E3N Access Committee. Further information is available at https://www.e3n.fr/node/78.
