# Supplementary material for: Adherence to the Mediterranean and Mediterranean‐Dietary Approaches to Stop Hypertension Intervention for Neurodegenerative Delay (MIND) Diets and Parkinson's Disease Incidence in Women: Results from the Prospective E3N Cohort
Source: Ann Neurol. 2026 Jan 6;99(4):1014–29. doi: 10.1002/ana.78115 (PMC13011782; doi:10.1002/ana.78115)
Supplement: Supplementary file 1 — Figure S1. Flow chart for inclusion into the study (5, 10, 15, and 20 years‐lags). Figure S2. Directed acyclic graph. Table S1. Participant's characteristics at baseline (1993‐Q3) according to Parkinson's disease status at the end of the follow‐up. Table S2. Frequency of consumption of individual components of the Mediterranean (MED) diet score. Table S3. Frequency of consumption of individuals components of the MIND diet score. Table S4. Association between the scores of adherence to the Mediterranean (MED) and MIND diet and PD incidence: analyses lagged by 10 years. Table S5. Association between the scores of adherence to the Mediterranean (MED) and MIND diet and PD incidence: analyses lagged by 15 years. Table S6. Association between the scores of adherence to the Mediterranean (MED) and MIND diet and PD incidence: analyses lagged by 20 years. Table S7. Association between the scores of adherence to the Mediterranean (MED) and MIND diet and PD incidence: analyses adjusted for constipation and depression. Table S8. Association between the scores of adherence to the modified Mediterranean (MED) diet (after exclusion of dairy products) and PD incidence. Table S9. Association between the scores of adherence to the Mediterranean (MED) and MIND diet and PD incidence: analyses adjusted for BMI, diabetes, and hypertension. Table S10. Association between the scores of adherence to the Mediterranean (MED) and MIND diet and PD incidence: analyses using the original scoring system for the MED and MIND diet scores. [file ANA-99-1014-s001.pdf]

Adherence to the Mediterranean and Mediterranean-Dietary Approaches to Stop Hypertension Intervention for Neurodegenerative Delay (MIND) diets and Parkinson's disease incidence in women: results from the prospective E3N cohort

Mariem Hajji-Louati, Emmanuelle Correia, Pei-Chen Lee, Fanny Artaud, Emmanuel Roze, Francesca Romana Mancini, Alexis Elbaz

Supplementary Figure S1. Flow chart for inclusion into the study (5y-, 10y-, 15y-, and 20y-lags). 2

Supplementary Figure S2. Directed acyclic graph. 3

Supplementary Table S1. Participant's characteristics at baseline (1993-Q3) according to Parkinson's disease status at the end of the follow-up. 4

Supplementary Table S2. Frequency of consumption of individual components of the Mediterranean (MED) diet score. 5

Supplementary Table S3. Frequency of consumption of individuals components of the MIND diet score. 6

Supplementary Table S4. Association between the scores of adherence to the Mediterranean (MED) and MIND diet and PD incidence: analyses lagged by 10 years.. 7

Supplementary Table S5. Association between the scores of adherence to the Mediterranean (MED) and MIND diet and PD incidence: analyses lagged by 15 years.. 8

Supplementary Table S6. Association between the scores of adherence to the Mediterranean (MED) and MIND diet and PD incidence: analyses lagged by 20 years.. 9

Supplementary Table S7. Association between the scores of adherence to the Mediterranean (MED) and MIND diet and PD incidence: analyses adjusted for constipation and depression. 10

Supplementary Table S8. Association between the scores of adherence to the modified Mediterranean (MED) diet (after exclusion of dairy products) and PD incidence. 11

Supplementary Table S9. Association between the scores of adherence to the Mediterranean (MED) and MIND diet and PD incidence: analyses adjusted for BMI, diabetes, and hypertension............................. 12

Supplementary Table S10. Association between the scores of adherence to the Mediterranean (MED) and MIND diet and PD incidence: analyses using the original scoring system for the MED and MIND diet scores. 13

Supplementary Figure S1. Flow chart for inclusion into the study (5y-, 10y-, 15y-, and 20y-lags).

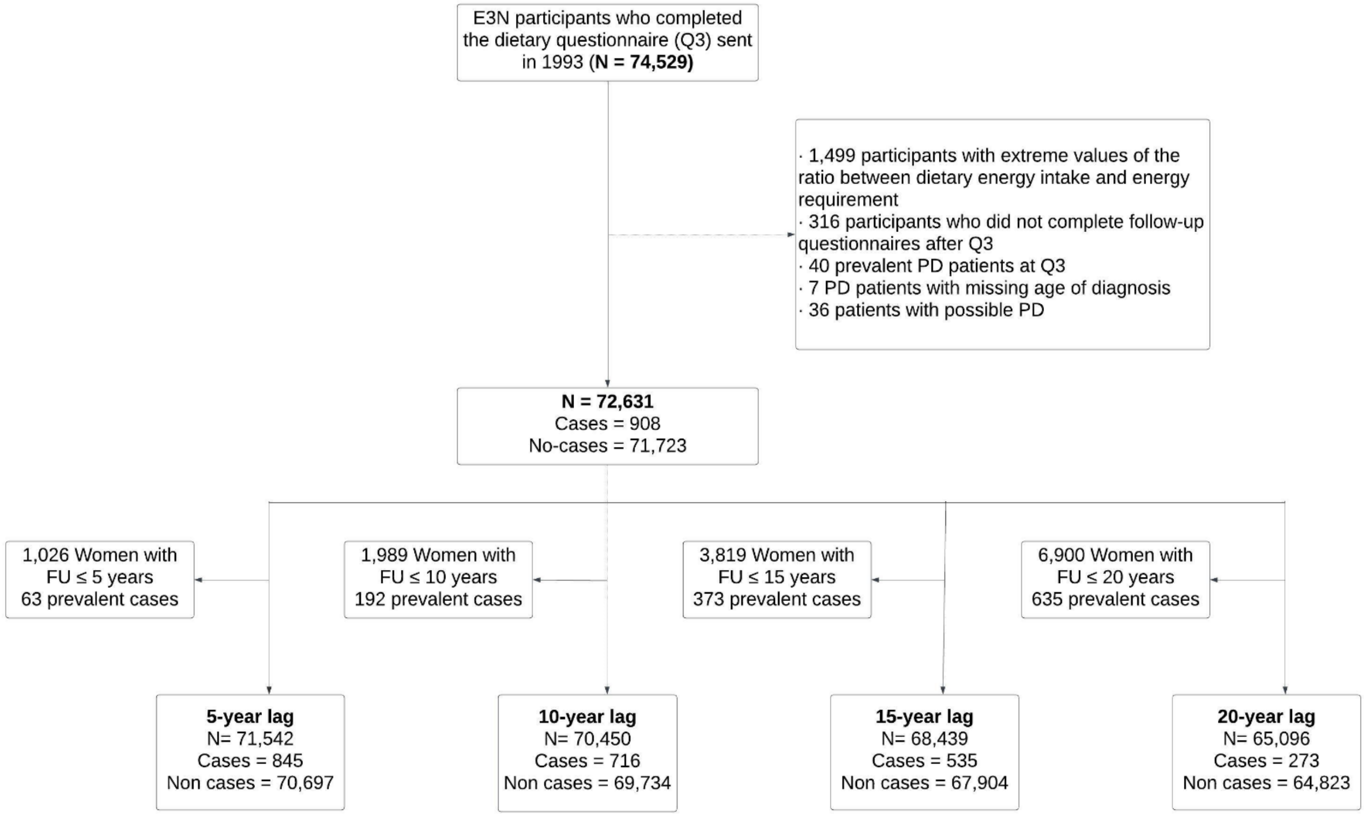

Supplementary Figure S2. Directed acyclic graph.

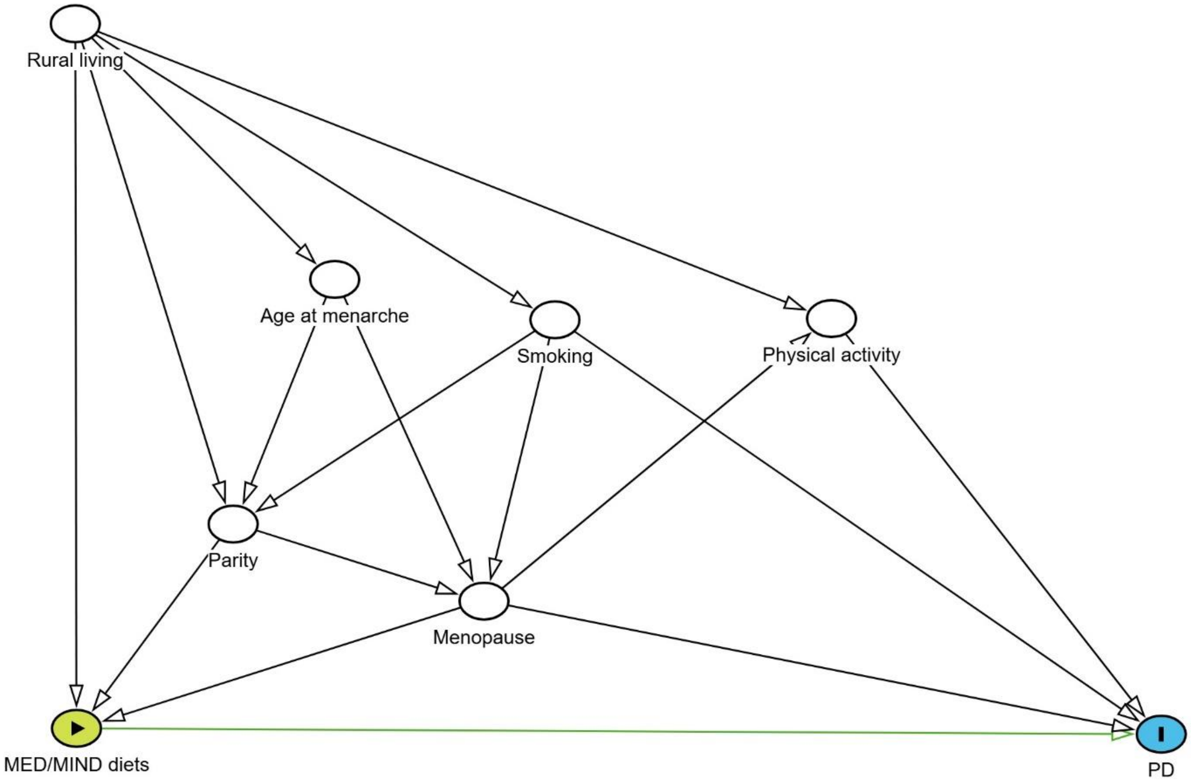

Supplementary Table S1. Participant's characteristics at baseline (1993-Q3) according to Parkinson's disease status at the end of the follow-up.

	**Parkinson's Disease**	
	**No**	**Yes**
**Characteristics, N (%)**	**N=70,697**	**N=845**
**Age (years), M (SD)**	52.8 (6.6)	56.1 (6.5)
**Rural residence**	13,325 (18.8)	149 (17.6)
**Menopausal status**	30,250 (42.8)	197 (23.3)
Premenopausal	30,250 (42.8)	197 (23.3)
Natural menopause	34,696 (49.1)	534 (63.2)
Artificial menopause	5,291 (7.5)	111 (13.1)
Unknown type of menopause	460 (0.7)	3 (0.4)
**Age at menarche (years)**		
≤11	14,389 (20.4)	177 (20.9)
12-13	36,683 (51.9)	407 (48.2)
≥14	19,625 (27.8)	261 (30.9)
**Parity**	8,357 (11.8)	93 (11.0)
Nulliparous	8,357 (11.8)	93 (11.0)
One child	11,080 (15.7)	107 (12.7)
Two children	30,790 (43.6)	353 (41.8)
≥three children	20,470 (29.0)	292 (34.6)
**Smoking**	37,882 (53.6)	478 (56.6)
Never	37,882 (53.6)	478 (56.6)
Ex	23,251 (32.9)	280 (33.1)
Current	9,564 (13.5)	87 (10.3)
**Body mass index (kg/m^2)**		
<18.5	2,947 (4.2)	28 (3.3)
18.5-24.9	56,613 (80.0)	671 (79.4)
25.0-29.9	9,376 (13.3)	132 (15.6)
≥30.0	1,761 (2.5)	14 (1.7)
**Hypertension**	26,293 (37.2)	350 (41.4)
**Diabetes**	814 (1.2)	10 (1.2)
**Physical activity (MET-h/week), M (SD)**	49.1 (46.0)	50.2 (42.6)
**Caffeine intake (mg/day), M (SD)**	202.2 (149.8)	191.5 (139.0)
**Adherence to Mediterranean diet, M (SD)**	3.9 (1.6)	3.9 (1.6)
**Total energy intake (Kcal/day), M (SD)**	2210.9 (560.4)	2204.2 (543.0)
**Constipation**	17,223 (24.4)	230 (27.2)
**Depression**	15,203 (21.5)	191 (22.6)

M, mean; SD, standard deviation; MET, metabolic equivalent of task.

Supplementary Table S2. Frequency of consumption of individual components of the Mediterranean (MED) diet score.

		Age at PD diagnosis		
**MED score component, N(%)**	**Overall**	**<71y**	**≥71y**	**P**
**Vegetables (g/day)**				
<182.4	17847 (24.9)	89 (27.7)	118 (22.5)	
182.4-349.9	35818 (50.1)	151 (47.0)	265 (50.6)	
≥349.9	17877 (25.0)	81 (25.2)	141 (26.9)	0.173
**Legumes (g/day)**				
<3.4	17850 (25)	94 (29.3)	146 (27.9)	
3.4-28.6	36011 (50.3)	155 (48.3)	276 (52.7)	
≥28.6	17681 (24.7)	72 (22.4)	102 (19.5)	0.754
**Fruits (g/day)**				
<138.6	18441 (25.8)	94 (29.3)	106 (20.2)	
138.6-330.7	35253 (49.3)	152 (47.4)	242 (46.2)	
≥330.7	17848 (24.9)	75 (23.4)	176 (33.6)	**<0.001**
**Cereals (g/day)**				
<119.1	17835 (24.9)	70 (21.8)	142 (27.1)	
119.1-246.6	35808 (50.1)	173 (53.9)	265 (50.6)	
≥246.6	17899 (25.0)	78 (24.3)	117 (22.3)	0.140
**Fish (g/day)**				
<12.4	11686 (16.3)	53 (16.5)	72 (13.7)	
12.4-37.3	44857 (62.7)	204 (63.6)	332 (63.4)	
≥37.3	14999 (21.0)	64 (19.9)	120 (22.9)	0.178
**Meat (g/day)**				
<67.9	17850 (25.0)	74 (23.1)	141 (26.9)	
67.9-132.2	35790 (50.0)	156 (48.6)	281 (53.6)	
≥132.2	17902 (25.0)	91 (28.3)	102 (19.5)	**0.010**
**Dairy product (g/day)**				
<117.9	18112 (25.3)	83 (25.9)	130 (24.8)	
117.9-342.2	35572 (49.7)	148 (46.1)	268 (51.1)	
≥342.2	17858 (25.0)	90 (28.0)	126 (24.0)	0.560
**Unsaturated fat:saturated fat ratio**				
<1.1	18372 (25.7)	93 (29.0)	142 (27.1)	
1.1-1.5	38289 (53.5)	177 (55.1)	262 (50.0)	
≥1.5	14881 (20.8)	51 (15.9)	120 (22.9)	0.069
**Alcohol (g/day)**				
<5	30440 (42.5)	128 (39.9)	229 (43.7)	
5_25	31406 (43.9)	143 (44.5)	230 (43.9)	
≥25	9696 (13.6)	50 (15.6)	65 (12.4)	0.153

Statistically significant p-values are bolded.

Supplementary Table S3. Frequency of consumption of individuals components of the MIND diet score.

MIND score component, N (%)	Overall	Age at PD diagnosis <71y	≥71y	P
**Whole grains**				
0 g/day	42060 (58.8)	178 (55.5)	302 (57.6)	
>0 g/day	29482 (41.2)	143 (44.5)	222 (42.4)	0.535
**Nuts**				
0 g/day	20175 (28.2)	80 (24.9)	181 (34.5)	
>0 g/day	51367 (71.8)	241 (75.1)	343 (65.5)	**0.003**
**Total polyphenols**				
< 1186 g/day	23598 (33.0)	90 (28.0)	186 (35.5)	
1186 - 1888 g/day	31470 (44.0)	150 (46.7)	232 (44.3)	
≥ 1888 g/day	16474 (23.0)	81 (25.2)	106 (20.2)	**0.017**
**Green leafy vegetables**				
<73 g/day	23586 (33.0)	109 (34.0)	150 (28.6)	
73-131 g/day	31433 (43.9)	137 (42.7)	233 (44.5)	
≥131 g/day	16523 (23.1)	75 (23.4)	141 (26.9)	0.094
**Olive oil**				
<median intake (3.3)	35848 (50.1)	178 (55.5)	242 (46.2)	
≥ median intake (3.3)	35694 (49.9)	143 (44.5)	282 (53.8)	**0.009**
**Other vegetables**				
< 7 times/week	1304 (1.8)	5 (1.6)	9 (1.7)	
[7-14]/week	10655 (14.9)	47 (14.6)	64 (12.2)	
>14 times/week	59583 (83.3)	269 (83.8)	451 (86.1)	0.472
**Beans**				
< 1 time/month	36507 (51.0)	174 (54.2)	288 (55.0)	
1/month-1/week	26013 (36.4)	113 (35.2)	185 (35.3)	
≥ 2 times/week	9022 (12.6)	34 (10.6)	51 (9.7)	0.734
**Fish**				
< 1 time/week	8368 (11.7)	35 (10.9)	49 (9.4)	
1 time/week	32951 (46.1)	153 (47.7)	242 (46.2)	
≥ 2 times/week	30223 (42.2)	133 (41.4)	233 (44.5)	0.319
**Poultry**				
< 1 time/week	23106 (32.3)	106 (33.0)	189 (36.1)	
1 time/week	43309 (60.5)	198 (61.7)	311 (59.4)	
≥ 2 times/week	5127 (7.2)	17 (5.3)	24 (4.6)	0.339
**Red meats and products**				
≥ 7 meals/week	10444 (14.6)	60 (18.7)	49 (9.4)	
5-6/week	32016 (44.8)	140 (43.6)	223 (42.6)	
≤ 4 meals/week	29082 (40.7)	121 (37.7)	252 (48.1)	**<0.001**
**Cheese**				
> 7 times / week	45457 (63.5)	212 (66.0)	328 (62.6)	
1-7/week	22829 (31.9)	97 (30.2)	161 (30.7)	
< 1 time/week	3256 (4.6)	12 (3.7)	35 (6.7)	0.130
**Pastries and sweets**				
≥ 7 times / week	38175 (53.4)	173 (53.9)	274 (52.3)	
5-6/week	7984 (11.2)	33 (10.3)	54 (10.3)	
< 5 times/week	25383 (35.5)	115 (35.8)	196 (37.4)	0.631
**Fried and fast food**				
> 2 times / week	762 (1.1)	5 (1.6)	3 (0.6)	
1-2/week	23030 (32.2)	96 (29.9)	128 (24.4)	
< 1 time/week	47750 (66.7)	220 (68.5)	393 (75.0)	**0.026**
**Butter and margarine**				
> 14 times/week	10155 (14.2)	54 (16.8)	76 (14.5)	
7-14/week	23168 (32.4)	112 (34.9)	147 (28.1)	
< 7 times/week	38219 (53.4)	155 (48.3)	301 (57.4)	**0.028**
**Wine**				
Never or >10 glasses/week	32777 (45.8)	145 (45.2)	238 (45.4)	
1/month-6/week	31292 (43.7)	149 (46.4)	229 (43.7)	
7-10 glasses/week	7473 (10.4)	27 (8.4)	57 (10.9)	0.632

Statistically significant p-values are bolded.

Supplementary Table S4. Association between the scores of adherence to the Mediterranean (MED) and MIND diet and PD incidence: analyses lagged by 10 years.

	Score of adherence to the MED diet						
**HR (95% CI)**	**Low**	**Medium**	**High**	**Per 1-unit**	**P-trend**	**Low/medium**	**High**
**Total population**							
N cases (IR per 100,000)	207 (71.7)	318 (71.3)	191 (75.8)	716 (72.6)		525 (71.5)	191 (75.8)
Model 1	1.00 (Reference)	0.97 (0.82-1.16)	1.00 (0.82-1.22)	0.99 (0.95-1.04)	0.667	1.00 (Reference)	1.02 (0.86-1.20)
Model 2	1.00 (Reference)	0.95 (0.80-1.14)	0.95 (0.78-1.16)	0.98 (0.93-1.02)	0.346	1.00 (Reference)	0.98 (0.83-1.16)
N cases (IR per 100,000)	79 (45.0)	111 (41.9)	50 (34.9)	240 (41.1)		190 (43.1)	50 (34.9)
Model 1	1.00 (Reference)	0.92 (0.69-1.23)	0.75 (0.53-1.07)	**0.92 (0.85-1.00)**	**0.039**	1.00 (Reference)	0.79 (0.58-1.08)
Model 2	1.00 (Reference)	0.89 (0.66-1.19)	0.71 (0.49-1.01)	**0.90 (0.83-0.98)**	**0.014**	1.00 (Reference)	0.76 (0.55-1.04)
**Age at PD diagnosis ≥ 71y**							
N cases (IR per 100,000)	128 (104.3)	207 (105.7)	141 (119.5)	476 (109.0)		335 (105.2)	141 (119.5)
Model 1	1.00 (Reference)	1.01 (0.81-1.26)	1.14 (0.90-1.45)	1.03 (0.97-1.09)	0.348	1.00 (Reference)	1.13 (0.93-1.38)
Model 2	1.00 (Reference)	0.99 (0.80-1.24)	1.10 (0.86-1.40)	1.02 (0.96-1.08)	0.571	1.00 (Reference)	1.10 (0.90-1.34)
	**Score of adherence to the MIND diet**						
	**Low**	**Medium**	**High**	**Per 1-unit**	**P-trend**	**Low/medium**	**High**
**Total population**							
N cases (IR per 100,000)	172 (68.3)	319 (73.5)	225 (74.7)	716 (72.6)		491 (71.6)	225 (74.7)
Model 1	1.00 (Reference)	1.03 (0.86-1.24)	1.02 (0.83-1.24)	1.01 (0.96-1.05)	0.777	1.00 (Reference)	1.00 (0.85-1.17)
Model 2	1.00 (Reference)	1.02 (0.85-1.23)	1.00 (0.82-1.23)	1.00 (0.96-1.05)	0.882	1.00 (Reference)	0.99 (0.84-1.16)
**Age at PD diagnosis < 71y**							
N cases (IR per 100,000)	69 (44.1)	110 (42.9)	61 (35.6)	240 (41.1)		179 (43.4)	61 (35.6)
Model 1	1.00 (Reference)	0.95 (0.70-1.28)	0.77 (0.54-1.08)	0.95 (0.88-1.03)	0.185	1.00 (Reference)	0.79 (0.59-1.06)
Model 2	1.00 (Reference)	0.94 (0.69-1.27)	0.76 (0.54-1.08)	0.95 (0.88-1.03)	0.182	1.00 (Reference)	0.79 (0.59-1.06)
**Age at PD diagnosis ≥ 71y**							
N cases (IR per 100,000)	103 (99.0)	209 (108.4)	164 (117.3)	476 (109.0)		312 (105.1)	164 (117.3)
Model 1	1.00 (Reference)	1.09 (0.86-1.38)	1.18 (0.92-1.51)	1.04 (0.98-1.10)	0.161	1.00 (Reference)	1.12 (0.92-1.35)
Model 2	1.00 (Reference)	1.08 (0.85-1.37)	1.17 (0.91-1.50)	1.04 (0.98-1.10)	0.209	1.00 (Reference)	1.11 (0.91-1.34)

IR, crude incidence rate of PD per 100,000 person-years; RCS, restricted cubic spline.

Hazard ratios (HR) and 95% confidence intervals (CI) calculated using Cox proportional hazards models with age as the time scale. Statistically significant HRs and p-values are bolded.

Model 1: adjusted for age (as the timescale).

Model 2: further adjusted for baseline place of residence (rural/urban), age at menarche (≤11/12-13/≥14 years), parity (nulliparous/one child/two children/≥three children), smoking (never/ex/current), menopausal status (premenopausal/natural/artificial/unknown type), physical activity (RCS-4 knots), caffeine intake (RCS-4 knots) and total energy intake (RCS-4 knots).

Supplementary Table S5. Association between the scores of adherence to the Mediterranean (MED) and MIND diet and PD incidence: analyses lagged by 15 years**.**

	Score of adherence to the MED diet						
**HR (95% CI)**	**Low**	**Medium**	**High**	**Per 1-unit**	**P-trend**	**Low/medium**	**High**
**Total population**							
N cases (IR per 100,000)	144 (77.1)	244 (84.5)	147 (90.2)	535 (83.8)		388 (81.6)	147 (90.2)
Model 1	1.00 (Reference)	1.08 (0.88-1.32)	1.11 (0.88-1.40)	1.02 (0.96-1.07)	0.549	1.00 (Reference)	1.06 (0.88-1.29)
Model 2	1.00 (Reference)	1.04 (0.85-1.28)	1.04 (0.83-1.32)	1.00 (0.95-1.06)	0.999	1.00 (Reference)	1.02 (0.84-1.23)
**Age at PD diagnosis < 71y**							
N cases (IR per 100,000)	46 (72.7)	76 (80.7)	29 (58.0)	151 (72.8)		122 (77.5)	29 (58.0)
Model 1	1.00 (Reference)	1.10 (0.76-1.59)	0.78 (0.49-1.24)	0.92 (0.83-1.02)	0.100	1.00 (Reference)	0.74 (0.49-1.10)
Model 2	1.00 (Reference)	1.06 (0.73-1.53)	0.71 (0.44-1.14)	**0.90 (0.81-0.99)**	**0.038**	1.00 (Reference)	0.69 (0.46-1.04)
**Age at PD diagnosis ≥ 71y**							
N cases (IR per 100,000)	98 (121.6)	168 (130.5)	118 (152.0)	384 (133.8)		266 (127.1)	118 (152.0)
Model 1	1.00 (Reference)	1.07 (0.84-1.38)	1.24 (0.95-1.62)	1.05 (0.99-1.12)	0.096	1.00 (Reference)	1.19 (0.96-1.48)
Model 2	1.00 (Reference)	1.04 (0.81-1.34)	1.17 (0.89-1.53)	1.04 (0.97-1.11)	0.253	1.00 (Reference)	1.14 (0.91-1.42)
	**Score of adherence to the MIND diet**						
	**Low**	**Medium**	**High**	**Per 1-unit**	**P-trend**	**Low/medium**	**High**
**Total population**							
N cases (IR per 100,000)	130 (79.8)	238 (84.7)	167 (85.8)	535 (83.8)		368 (82.9)	167 (85.8)
Model 1	1.00 (Reference)	1.02 (0.82-1.26)	1.00 (0.80-1.26)	1.00 (0.95-1.05)	0.951	1.00 (Reference)	0.99 (0.82-1.19)
Model 2	1.00 (Reference)	1.01 (0.81-1.25)	0.99 (0.78-1.25)	1.00 (0.94-1.05)	0.861	1.00 (Reference)	0.98 (0.82-1.18)
**Age at PD diagnosis < 71y**							
N cases (IR per 100,000)	49 (85.8)	70 (76.8)	32 (54.1)	151 (72.8)		119 (80.2)	32 (54.1)
Model 1	1.00 (Reference)	0.88 (0.61-1.26)	0.61 (0.39-0.95)	**0.89 (0.81-0.98)**	**0.021**	1.00 (Reference)	**0.66 (0.44-0.97)**
Model 2	1.00 (Reference)	0.88 (0.61-1.27)	0.61 (0.39-0.96)	**0.89 (0.81-0.99)**	**0.025**	1.00 (Reference)	**0.66 (0.45-0.98)**
**Age at PD diagnosis ≥ 71y**							
N cases (IR per 100,000)	81 (118.6)	168 (132.7)	135 (146.7)	384 (133.8)		249 (127.8)	135 (146.7)
Model 1	1.00 (Reference)	1.12 (0.86-1.46)	1.24 (0.94-1.63)	1.05 (0.99-1.12)	0.113	1.00 (Reference)	1.15 (0.93-1.42)
Model 2	1.00 (Reference)	1.10 (0.84-1.44)	1.21 (0.91-1.60)	1.04 (0.98-1.11)	0.177	1.00 (Reference)	1.13 (0.91-1.40)

IR, crude incidence rate of PD per 100,000 person-years; RCS, restricted cubic spline.

Hazard ratios (HR) and 95% confidence intervals (CI) calculated using Cox proportional hazards models with age as the time scale.

Model 1: adjusted for age (as the timescale).

Model 2: further adjusted for baseline place of residence (rural/urban), age at menarche (≤11/12-13/≥14 years), parity (nulliparous/one child/two children/≥three children), smoking (never/ex/current), menopausal status (premenopausal/natural/artificial/unknown type), physical activity (RCS-4 knots), caffeine intake (RCS-4 knots) and total energy intake (RCS-4 knots).

Supplementary Table S6. Association between the scores of adherence to the Mediterranean (MED) and MIND diet and PD incidence: analyses lagged by 20 years.

HR (95% CI)	Low	Medium	High	Per 1-unit	P-trend	Low/medium	High
	**Score of adherence to the MED diet**						
**Total population**							
N cases (IR per 100,000)	77 (86.4)	118 (85.8)	78 (101.1)	273 (89.9)		195 (86.1)	78 (101.1)
Model 1	1.00 (Reference)	0.97 (0.73-1.30)	1.11 (0.81-1.52)	1.02 (0.95-1.09)	0.644	1.00 (Reference)	1.13 (0.87-1.47)
Model 2	1.00 (Reference)	0.95 (0.71-1.27)	1.04 (0.75-1.44)	1.00 (0.93-1.08)	0.975	1.00 (Reference)	1.08 (0.82-1.40)
**Age at PD diagnosis < 71y**							
N cases (IR per 100,000)	15 (87.5)	26 (103.8)	8 (62.0)	49 (88.9)		41 (97.2)	8 (62.0)
Model 1	1.00 (Reference)	1.18 (0.63-2.23)	0.69 (0.29-1.64)	0.89 (0.75-1.06)	0.179	1.00 (Reference)	0.63 (0.29-1.34)
Model 2	1.00 (Reference)	1.13 (0.59-2.14)	0.63 (0.26-1.51)	0.86 (0.72-1.03)	0.102	1.00 (Reference)	0.59 (0.27-1.26)
**Age at PD diagnosis ≥ 71y**							
N cases (IR per 100,000)	62 (107.6)	92 (99.8)	70 (125.8)	224 (109.0)		154 (102.8)	70 (125.8)
Model 1	1.00 (Reference)	0.93 (0.67-1.28)	1.16 (0.83-1.64)	1.04 (0.96-1.13)	0.350	1.00 (Reference)	1.21 (0.92-1.61)
Model 2	1.00 (Reference)	0.90 (0.65-1.25)	1.09 (0.77-1.55)	1.02 (0.94-1.11)	0.588	1.00 (Reference)	1.16 (0.87-1.55)
	**Score of adherence to the MIND diet**						
	**Low**	**Medium**	**High**	**Per 1-unit**	**P-trend**	**Low/medium**	**High**
**Total population**							
N cases (IR per 100,000)	63 (81.1)	120 (89.6)	90 (97.6)	273 (89.9)		183 (86.5)	90 (97.6)
Model 1	1.00 (Reference)	1.06 (0.78-1.44)	1.12 (0.81-1.54)	1.03 (0.96-1.11)	0.411	1.00 (Reference)	1.07 (0.83-1.38)
Model 2	1.00 (Reference)	1.07 (0.79-1.46)	1.15 (0.83-1.59)	1.04 (0.96-1.12)	0.318	1.00 (Reference)	1.10 (0.85-1.42)
**Age at PD diagnosis < 71y**							
N cases (IR per 100,000)	15 (95.0)	23 (94.7)	11 (73.3)	49 (88.9)		38 (94.8)	11 (73.3)
Model 1	1.00 (Reference)	0.98 (0.51-1.89)	0.75 (0.34-1.63)	0.94 (0.79-1.11)	0.455	1.00 (Reference)	0.75 (0.39-1.48)
Model 2	1.00 (Reference)	1.02 (0.53-1.97)	0.83 (0.37-1.83)	0.96 (0.81-1.15)	0.655	1.00 (Reference)	0.82 (0.41-1.61)
**Age at PD diagnosis ≥ 71y**							
N cases (IR per 100,000)	48 (99.0)	97 (106.9)	79 (119.3)	224 (109.0)		145 (104.1)	79 (119.3)
Model 1	1.00 (Reference)	1.08 (0.76-1.53)	1.21 (0.85-1.74)	1.05 (0.97-1.14)	0.192	1.00 (Reference)	1.15 (0.88-1.52)
Model 2	1.00 (Reference)	1.09 (0.77-1.54)	1.23 (0.85-1.77)	1.06 (0.97-1.15)	0.176	1.00 (Reference)	1.16 (0.88-1.54)

IR, crude incidence rate of PD per 100,000 person-years; RCS, restricted cubic spline.
Hazard ratios (HR) and 95% confidence intervals (CI) calculated using Cox proportional hazards models with age as the time scale.
Model 1: adjusted for age (as the timescale).
Model 2: further adjusted for baseline place of residence (rural/urban), age at menarche (≤11/12-13/≥14 years), parity (nulliparous/one child/two children/≥three children), smoking (never/ex/current), menopausal status (premenopausal/natural/artificial/unknown type), physical activity (RCS-4 knots), caffeine intake (RCS-4 knots) and total energy intake (RCS-4 knots).

Supplementary Table S7. Association between the scores of adherence to the Mediterranean (MED) and MIND diet and PD incidence: analyses adjusted for constipation and depression.

	5y-lag		10y-lag		15y-lag		20y-lag	
**Dietary scores**	**HR (95% CI)**	**P-value**	**HR (95% CI)**	**P-value**	**HR (95% CI)**	**P-value**	**HR (95% CI)**	**P-value**
**Score of adherence to the MED diet**								
**Total population**								
Low/medium	1.00 (Reference)		1.00 (Reference)		1.00 (Reference)		1.00 (Reference)	
High	0.96 (0.82-1.12)	0.600	0.98 (0.83-1.16)	0.850	1.02 (0.84-1.24)	0.845	1.08 (0.83-1.41)	0.570
Per 1-unit	0.99 (0.94-1.03)	0.507	0.98 (0.93-1.03)	0.359	1.00 (0.95-1.06)	0.959	1.00 (0.93-1.08)	0.940
**Age at PD diagnosis < 71y**								
Low/medium	1.00 (Reference)		1.00 (Reference)		1.00 (Reference)		1.00 (Reference)	
High	0.77 (0.59-1.00)	0.054	0.76 (0.56-1.04)	0.089	0.69 (0.46-1.04)	0.079	0.59 (0.28-1.27)	0.176
Per 1-unit	**0.93 (0.86-0.99)**	**0.031**	**0.91 (0.84-0.98)**	**0.015**	**0.90 (0.81-1.00)**	**0.042**	0.86 (0.72-1.03)	0.109
**Age at PD diagnosis ≥ 71y**								
Low/medium	1.00 (Reference)		1.00 (Reference)		1.00 (Reference)		1.00 (Reference)	
High	1.09 (0.90-1.32)	0.393	1.10 (0.90-1.34)	0.347	1.14 (0.91-1.42)	0.243	1.17 (0.87-1.55)	0.295
Per 1-unit	1.02 (0.97-1.08)	0.390	1.02 (0.96-1.08)	0.562	1.04 (0.98-1.11)	0.238	1.02 (0.94-1.11)	0.566
**Score of adherence to the MIND diet**								
**Total population**								
Low/medium	1.00 (Reference)		1.00 (Reference)		1.00 (Reference)		1.00 (Reference)	
High	0.94 (0.81-1.09)	0.378	0.99 (0.84-1.16)	0.916	0.98 (0.82-1.18)	0.860	1.10 (0.85-1.42)	0.477
Per 1-unit	1.00 (0.96-1.04)	0.926	1.00 (0.96-1.05)	0.878	1.00 (0.94-1.05)	0.871	1.04 (0.96-1.12)	0.314
**Age at PD diagnosis < 71y**								
Low/medium	1.00 (Reference)		1.00 (Reference)		1.00 (Reference)		1.00 (Reference)	
High	**0.76 (0.59-0.98)**	**0.031**	0.79 (0.59-1.06)	0.122	**0.66 (0.45-0.98)**	**0.041**	0.82 (0.41-1.61)	0.563
Per 1-unit	0.96 (0.90-1.03)	0.294	0.95 (0.88-1.03)	0.185	**0.89 (0.81-0.99)**	**0.026**	0.96 (0.81-1.15)	0.662
**Age at PD diagnosis ≥ 71y**								
Low/medium	1.00 (Reference)		1.00 (Reference)		1.00 (Reference)		1.00 (Reference)	
High	1.06 (0.88-1.27)	0.542	1.11 (0.91-1.34)	0.297	1.13 (0.92-1.40)	0.251	1.17 (0.88-1.54)	0.281
Per 1-unit	1.02 (0.97-1.08)	0.441	1.04 (0.98-1.10)	0.208	1.04 (0.98-1.11)	0.173	1.06 (0.98-1.15)	0.175

RCS, restricted cubic spline.

Hazard ratios (HR) and 95% confidence intervals (CI) calculated using Cox proportional hazards models with age as the time scale. Statistically significant HRs and p-values are bolded.

Model adjusted for age (as the timescale), baseline place of residence (rural/urban), age at menarche (≤11/12-13/≥14 years), parity (nulliparous/one child/two children/≥three children), smoking (never/ex/current), menopausal status (premenopausal/natural/artificial/unknown type), physical activity (RCS-4 knots), caffeine intake (RCS-4 knots), total energy intake (RCS-4 knots), depression (no/yes) and constipation (no/yes).

Supplementary Table S8. Association between the scores of adherence to the modified Mediterranean (MED) diet (after exclusion of dairy products) and PD incidence.

	Score of adherence to the MED diet								
**HR (95% CI)**	**Low**	**Medium**	**High**	**Per 1-unit**	**P-trend**	**P-interaction[a]**	**Low/medium**	**High**	**P-interaction[b]**
**Total population**									
N cases (IR per 100,000)	332 (60.8)	381 (65.2)	132 (62.3)	845 (63.0)	--	--	713 (63.1)	132 (62.3)	--
Model 1	1.00 (Reference)	1.03 (0.89-1.20)	0.96 (0.78-1.17)	1.00 (0.95-1.04)	0.846	--	1.00 (Reference)	0.94 (0.78-1.13)	--
Model 2	1.00 (Reference)	1.00 (0.86-1.16)	0.90 (0.74-1.11)	0.98 (0.94-1.03)	0.412	--	1.00 (Reference)	0.90 (0.75-1.09)	--
**Age at PD diagnosis < 71y**									
N cases (IR per 100,000)	136 (54.0)	150 (57.4)	35 (37.9)	321 (53.0)	--	--	286 (55.7)	35 (37.9)	--
Model 1	1.00 (Reference)	1.04 (0.83-1.32)	**0.68 (0.47-0.98)**	0.95 (0.88-1.02)	0.138	--	1.00 (Reference)	**0.66 (0.47-0.94)**	--
Model 2	1.00 (Reference)	0.98 (0.78-1.25)	**0.61 (0.42-0.89)**	**0.92 (0.86-0.99)**	**0.028**	--	1.00 (Reference)	**0.62 (0.43-0.88)**	--
**Age at PD diagnosis ≥ 71y**									
N cases (IR per 100,000)	196 (173.3)	231 (178.5)	97 (196.8)	524 (179.6)	--	--	427 (176.1)	97 (196.8)	--
Model 1	1.00 (Reference)	1.03 (0.85-1.24)	1.13 (0.89-1.44)	1.03 (0.97-1.08)	0.360	0.083	1.00 (Reference)	1.11 (0.89-1.39)	**0.015**
Model 2	1.00 (Reference)	1.02 (0.84-1.23)	1.11 (0.86-1.42)	1.02 (0.96-1.08)	0.481	**0.030**	1.00 (Reference)	1.10 (0.88-1.37)	**0.007**

IR, crude incidence rate per 100,000 person-years; RCS, restricted cubic spline.
Hazard ratios (HR) and 95% confidence intervals (CI) calculated using Cox proportional hazards models with age as the time scale. Statistically significant HRs and p-values are bolded.
Model 1: adjusted for age (as the timescale).
Model 2: further adjusted for baseline place of residence (rural/urban), age at menarche (≤11/12-13/≥14y), parity (nulliparous/one child/two children/≥three children), smoking (never/ex/current), menopausal status (premenopausal/natural/artificial/unknown type), physical activity (RCS-4 knots), caffeine intake (RCS-4 knots) and total energy intake (RCS-4 knots) and plain milk consumption (no/yes).
[a] P-value for the difference in HRs per 1-unit between the two age strata.
[b] P-value for the difference in HRs comparing the high vs. low+medium groups between the two age strata.

Supplementary Table S9. Association between the scores of adherence to the Mediterranean (MED) and MIND diet and PD incidence: analyses adjusted for BMI, diabetes, and hypertension.

	**Score of adherence to the MED diet**								
**HR (95% CI)**	**Low**	**Medium**	**High**	**Per 1-unit**	**P-trend**	**P-interaction[a]**	**Low/medium**	**High**	**P-interaction[b]**
**Total population**									
N cases (IR per 100,000)	236 (60.1)	388 (64.0)	221 (64.4)	845 (63.0)	--	--	624 (62.5)	221 (64.4)	--
Model 1	1.00 (Reference)	1.04 (0.89-1.23)	1.01 (0.84-1.22)	1.00 (0.96-1.04)	0.845	--	1.00 (Reference)	0.99 (0.85-1.15)	--
Model 2	1.00 (Reference)	1.02 (0.87-1.20)	0.97 (0.80-1.17)	0.98 (0.94-1.03)	0.462	--	1.00 (Reference)	0.96 (0.82-1.12)	--
**Age at PD diagnosis < 71y**									
N cases (IR per 100,000)	97 (35.8)	155 (37.6)	69 (30.6)	321 (35.3)	--	--	252 (36.9)	69 (30.6)	--
Model 1	1.00 (Reference)	1.04 (0.81-1.34)	0.83 (0.61-1.13)	0.95 (0.88-1.01)	0.106	--	1.00 (Reference)	0.81 (0.62-1.06)	--
Model 2	1.00 (Reference)	1.00 (0.77-1.29)	0.76 (0.56-1.04)	**0.93 (0.86-0.99)**	**0.028**	--	1.00 (Reference)	**0.76 (0.58-1.00)**	--
**Age at PD diagnosis ≥ 71y**									
N cases (IR per 100,000)	139 (113.0)	233 (118.6)	152 (128.6)	524 (119.7)	--	--	372 (116.4)	152 (128.6)	--
Model 1	1.00 (Reference)	1.05 (0.85-1.29)	1.13 (0.90-1.43)	1.03 (0.97-1.08)	0.309	0.058	1.00 (Reference)	1.10 (0.91-1.33)	0.065
Model 2	1.00 (Reference)	1.04 (0.84-1.29)	1.11 (0.88-1.41)	1.02 (0.97-1.08)	0.418	**0.026**	1.00 (Reference)	1.08 (0.89-1.31)	**0.038**
	**Score of adherence to the MIND diet**								
	**Low**	**Medium**	**High**	**Per 1-unit**	**P-trend**		**Low/medium**	**High**	
**Total population**									
N cases (IR per 100,000)	200 (58.4)	390 (67.0)	255 (66.1)	845 (62.3)	--	--	590 (63.3)	255 (62.3)	--
Model 1	1.00 (Reference)	1.09 (0.92-1.29)	1.00 (0.83-1.20)	1.00 (0.96-1.04)	0.949	--	1.00 (Reference)	0.94 (0.82-1.09)	--
Model 2	1.00 (Reference)	1.07 (0.91-1.28)	0.98 (0.81-1.18)	1.00 (0.96-1.04)	0.885	--	1.00 (Reference)	0.93 (0.80-1.08)	--
**Age at PD diagnosis < 71y**									
N cases (IR per 100,000)	82 (34.3)	158 (39.7)	81 (29.9)	321 (35.3)	--	--	240 (37.6)	81 (29.9)	--
Model 1	1.00 (Reference)	1.13 (0.87-1.48)	0.84 (0.61-1.14)	0.97 (0.91-1.04)	0.397	--	1.00 (Reference)	**0.77 (0.60-0.99)**	--
Model 2	1.00 (Reference)	1.10 (0.84-1.44)	0.81 (0.59-1.10)	0.96 (0.90-1.03)	0.287	--	1.00 (Reference)	**0.75 (0.59-0.97)**	--
**Age at PD diagnosis ≥ 71y**									
N cases (IR per 100,000)	118 (113.0)	232 (120.0)	174 (124.3)	524 (119.7)	--	--	350 (117.6)	174 (124.3)	--
Model 1	1.00 (Reference)	1.06 (0.85-1.32)	1.10 (0.87-1.38)	1.02 (0.97-1.08)	0.446	0.255	1.00 (Reference)	1.06 (0.88-1.27)	**0.045**
Model 2	1.00 (Reference)	1.06 (0.85-1.32)	1.10 (0.86-1.39)	1.02 (0.97-1.08)	0.463	0.197	1.00 (Reference)	1.05 (0.88-1.27)	**0.038**

IR, crude incidence rate per 100,000 person-years; RCS, restricted cubic spline.

Hazard ratios (HR) and 95% confidence intervals (CI) calculated using Cox proportional hazards models with age as the time scale. Statistically significant HRs and p-values are bolded.

Model 1: adjusted for age (as the timescale).

Model 2: further adjusted for baseline place of residence (rural/urban), age at menarche (≤11/12-13/≥14y), parity (nulliparous/one child/two children/≥three children), smoking (never/ex/current), menopausal status (premenopausal/natural/artificial/unknown type), physical activity (RCS-4 knots), BMI (<18.5, 18.5-24.9, 25.0-29.9, ≥30 kg/m²), hypertension (no/yes), diabetes (no/yes), caffeine intake (RCS-4 knots) and total energy intake (RCS-4 knots).

[a] P-value for the difference in HRs per 1-unit between the two age strata.

[b] P-value for the difference in HRs comparing the high vs. low+medium groups between the two age strata.

Supplementary Table S10. Association between the scores of adherence to the Mediterranean (MED) and MIND diet and PD incidence: analyses using the original scoring system for the MED and MIND diet scores.

	Score of adherence to the MED diet								
**HR (95% CI)**	**Low**	**Medium**	**High**	**Per 1-unit**	**P-trend**	**P-interaction[a]**	**Low/medium**	**High**	**P-interaction[b]**
**Total population**									
N cases (IR per 100,000)	250 (60.1)	380 (64.5)	215 (63.8)	845 (63.0)	--	--	630 (62.7)	215 (63.8)	--
Model 1	1.00 (Reference)	1.05 (0.89-1.23)	1.00 (0.83-1.19)	1.00 (0.96-1.04)	0.938	--	1.00 (Reference)	0.97 (0.83-1.13)	--
Model 2	1.00 (Reference)	1.02 (0.87-1.20)	0.95 (0.78-1.14)	0.99 (0.95-1.03)	0.530	--	1.00 (Reference)	0.94 (0.80-1.10)	--
**Age at PD diagnosis < 71y**									
N cases (IR per 100,000)	100 (34.7)	156 (39.1)	65 (29.4)	321 (35.3)	--	--	256 (37.2)	65 (29.4)	--
Model 1	1.00 (Reference)	1.11 (0.86-1.42)	0.82 (0.60-1.12)	0.96 (0.90-1.02)	0.196	--	1.00 (Reference)	0.77 (0.59-1.01)	--
Model 2	1.00 (Reference)	1.05 (0.82-1.36)	0.74 (0.54-1.02)	**0.93 (0.87-1.00)**	**0.047**	--	1.00 (Reference)	**0.72 (0.54-0.95)**	--
**Age at PD diagnosis ≥ 71y**									
N cases (IR per 100,000)	150 (116.1)	224 (117.0)	150 (128.1)	524 (119.7)	--	--	374 (116.6)	150 (128.1)	--
Model 1	1.00 (Reference)	1.01 (0.82-1.24)	1.10 (0.88-1.38)	1.02 (0.97-1.08)	0.359	0.113	1.00 (Reference)	1.09 (0.90-1.32)	**0.038**
Model 2	1.00 (Reference)	1.00 (0.81-1.23)	1.08 (0.85-1.37)	1.02 (0.97-1.08)	0.459	**0.044**	1.00 (Reference)	1.08 (0.89-1.31)	**0.017**
	**Score of adherence to the MIND diet**								
	**Low**	**Medium**	**High**	**Per 1-unit**	**P-trend**		**Low/medium**	**High**	
**Total population**									
N cases (IR per 100,000)	194 (55.9)	381 (65.0)	270 (66.0)	845 (63.0)	--	--	575 (61.6)	270 (66.0)	--
Model 1	1.00 (Reference)	1.08 (0.91-1.29)	1.04 (0.86-1.25)	1.00 (0.96-1.04)	0.916	--	1.00 (Reference)	0.99 (0.85-1.14)	--
Model 2	1.00 (Reference)	1.08 (0.91-1.28)	1.03 (0.85-1.24)	1.00 (0.96-1.04)	0.979	--	1.00 (Reference)	0.98 (0.85-1.13)	--
**Age at PD diagnosis < 71y**									
N cases (IR per 100,000)	88 (35.5)	157 (39.6)	76 (28.9)	321 (35.3)	--	--	245 (38.0)	76 (28.9)	--
Model 1	1.00 (Reference)	1.08 (0.83-1.40)	0.76 (0.56-1.04)	0.96 (0.90-1.02)	0.219	--	1.00 (Reference)	**0.73 (0.56-0.94)**	--
Model 2	1.00 (Reference)	1.07 (0.82-1.39)	0.75 (0.55-1.03)	0.96 (0.90-1.02)	0.194	--	1.00 (Reference)	**0.72 (0.56-0.94)**	--
**Age at PD diagnosis ≥ 71y**									
N cases (IR per 100,000)	106 (106.3)	224 (117.2)	194 (132.1)	524 (119.7)	--	--	330 (113.5)	194 (132.1)	--
Model 1	1.00 (Reference)	1.10 (0.87-1.38)	1.23 (0.97-1.56)	1.03 (0.98-1.08)	0.270	0.099	1.00 (Reference)	1.16 (0.97-1.38)	**0.004**
Model 2	1.00 (Reference)	1.09 (0.86-1.37)	1.22 (0.96-1.55)	1.03 (0.97-1.08)	0.329	0.104	1.00 (Reference)	1.15 (0.96-1.37)	**0.004**

IR, crude incidence rate of PD per 100,000 person-years; RCS, restricted cubic spline.

Hazard ratios (HR) and 95% confidence intervals (CI) calculated using Cox proportional hazards models with age as the time scale.

Model 1: adjusted for age (as the timescale).

Model 2: further adjusted for baseline place of residence (rural/urban), age at menarche (≤11/12-13/≥14 years), parity (nulliparous/one child/two children/≥three children), smoking (never/ex/current), menopausal status (premenopausal/natural/artificial/unknown type), physical activity (RCS-4 knots), caffeine intake (RCS-4 knots) and total energy intake (RCS-4 knots).
